# Telmisartan Repurposing Targets Novel Biomarkers for Precision Colorectal Cancer Therapy

**DOI:** 10.3390/pharmaceutics18081029

**Published:** 2026-08-20

**Authors:** Sarah Hunachagi, Hoor Hashim Alqudihi, Sayed AbdulAzeez, J. Francis Borgio, Dana Almohazey

**Affiliations:** 1 Masters of Science in Biotechnology Program, Institute for Research and Medical Consultations (IRMC), Imam Abdulrahman Bin Faisal University, Dammam 31441, Saudi Arabia; 2 Department of Genetic Research, Institute for Research and Medical Consultations (IRMC), Imam Abdulrahman Bin Faisal University, Dammam 31441, Saudi Arabia; fbalexander@iau.edu.sa; 3 Department of Epidemic Diseases Research, Institute for Research and Medical Consultations (IRMC), Imam Abdulrahman Bin Faisal University, Dammam 31441, Saudi Arabia; 4 Department of Stem Cell Research, Institute for Research and Medical Consultations (IRMC), Imam Abdulrahman Bin Faisal University, Dammam 31441, Saudi Arabia

**Keywords:** colorectal cancer, drug repurposing, *in-silico* analysis, *CLUH*, *CLSTN3*, *SLC11A2*, Telmisartan

## Abstract

**Background/Objectives**: Colorectal cancer (CRC) remains a leading cause of cancer-associated mortality worldwide. The current therapeutic interventions are heavily constrained by the development of resistance and severe systemic toxicity. To address these challenges, this study integrated a multi-disciplinary framework involving high-throughput in silico screening followed by in vitro experimental validation to identify novel genetic targets of CRC and evaluate the efficacy of FDA-approved drugs. The primary objective was to identify safe and selective therapeutic agents capable of modulating their effect. **Methods**: The methodology employed a systematic screening of recent large-scale Genome-Wide Association Studies (GWASs) to pinpoint novel targets, followed by in silico pathogenicity prediction, homology modelling and high-throughput virtual screening of over 1615 FDA-approved drugs. The prioritized candidates were validated in vitro using MTT cytotoxicity assays and differential gene expression analysis across CRC cell lines (HCT116 and HT29) and a non-tumorigenic control, Human embryonic kidney cell line HEK293. **Results**: In silico analysis identified *CLUH*, *CLSTN3* and *SLC11A2* as novel potential targets. Based on in silico predicted deleterious mutations and subsequent molecular docking-based virtual screening, Telmisartan, Dutasteride and Venetoclax were prioritized. This prioritization was supported by their high binding affinity and dose-dependent cytotoxicity in MTT assays; thus, suggesting their repurposing potential for CRC treatment. Telmisartan exhibited a superior therapeutic profile not only in terms of the statistically significant cytotoxicity (*p* < 0.01), but also its selective effect on HCT116 and HT29 when compared to high safety profile in HEK293. This was further validated when Telmisartan selectively downregulated *CLUH* and *SLC11A2* in CRC cell lines, HCT116 and HT29 while maintaining expression levels in the non-cancerous HEK293 cell line remained significantly unaffected. Furthermore, a 100 ns molecular dynamics simulation confirmed the stable binding conformation and structural reliability of the SLC11A2 (Trp179Ser)–Telmisartan complex. **Conclucions**: Our findings conclude that Telmisartan is a promising candidate for drug repurposing for CRC treatment and capable of modulating selected novel biomarkers *CLUH* and *SLC11A2*. However, further multi-omics-based confirmatory studies and pre-clinical validation studies are needed in the future to confirm the long-term efficacy of this repositioning strategy.

## 1. Introduction

Cancer is one of the most rapidly growing and deadly conditions globally. It has captured the attention of researchers for decades, yet till date raises many questions about its complex cellular and molecular pathology. It arises from a series of multifactorial events involving interactions between genetic and environmental factors, a process known as carcinogenesis, tumorigenesis or oncogenesis [1,2,3]. Carcinogenesis is a multistage process that involves mutations in a series of genes responsible for maintaining cellular homeostasis [4]. Identifying these genes and their related proteins is crucial for understanding the pathogenesis of malignant transformation, cancer diagnosis, prognosis and evaluating the potential success of treatment [5,6,7,8].

Colorectal cancer affects about 1.9 million people every year and has a notable genetic predisposition [9,10,11]. It ranks as the third most prevalent cancer and second leading cause of cancer-associated deaths worldwide [12,13,14,15]. This approximately accounts for 9.2% of total deaths globally [16,17,18]. Regardless of improving survival rates, metastatic colorectal cancer (mCRC) continues to be highly fatal, with a 5-year survival rate of only 14% [12,13]. Males have a 25% greater chance of developing CRC as compared to females [17,18]. In addition, age plays a crucial role in increasing the risk of CRC [19]. Moreover, recent trends in rectal and distal colon cancers have shown an increased early-onset CRC incidence (under the age of 50). This shift in onset age could be influenced by the global changes in factors like Western diet, stress and use of antibiotics [18,20].

The etiology of CRC is still a subject of study. However, research has shown that CRC is influenced by a number of factors. These include genetic factors (e.g., Familial Adenomatous Polyposis), dietary factors (e.g., high-fat and low-cellulose diet) and chronic inflammations (e.g., Crohn’s disease and ulcerative colitis) [21,22,23]. Other factors can also increase the risk of developing CRC such as a sedentary lifestyle, overweight and exposure to carcinogenic chemicals [19].

Histopathological changes in the intestine begin with epithelial hyperplasia followed by atypical hyperplasia, adenoma formation, localized carcinoma and invasive carcinoma [19,24]. Mutations in target oncogenes, tumor suppressor genes and the genes linked to DNA repair mechanism will result in CRC. Based on the source of mutation, CRC can be categorized as sporadic (70%), inherited (5%) and familial (25%) [25]. CRC develops through multiple well-differentiated molecular pathways [26]. Studies have revealed three pathways that are involved in developing these types of CRC: chromosomal instability (CIN), microsatellite instability (MSI) and the CpG island methylator phenotype (CIMP). Major signaling pathways including WNT, MAPK/PI3K, TGF-β and P53 are disrupted because of these mutations [25]. Moreover, these processes are frequently linked to irregular changes in several genes, including *APC*, *DCC*, *TP53*, *KRAS*, *c-MYC*, *MCC* and genes related to DNA mismatch repair (*hMLH1*, *hMLH3*, *hMSH2*, *hMSH3*, *hMSH6*, *hPMS1* and *hPMS2*) [19,27,28].

Chromosomal instability (CIN) is characterized by significant alterations in chromosome number and structure by deletions, insertions, translocations and so on. Particularly, CIN has been reported to cause inactivation of the *APC* tumor suppressor gene. CIN is a hallmark of majority of the sporadic CRC tumors (~84%) that develop by the adenoma-carcinoma sequence [29]. The second group, comprising approximately 13–16% of sporadic CRC, is characterized by hypermutation and MSI resulting from defective DNA mismatch repair (dMMR). This group is often associated with wild-type *TP53* and displays a near-diploid pattern of chromosomal instability. Additionally, epigenetic instability induced by CIMP is a result of promoter hypermethylation that leads to silencing of various tumor suppressor genes and *dMMR* genes [30,31,32].

Current CRC treatments have some limitations. While the traditional chemotherapeutic agents are highly effective, they are DNA-damaging and cause significant toxicity that limit their long-term use. In addition, tumor cells eventually develop resistance to these drugs, necessitating additional lines of treatment [13]. These limitations highlight the urgent need for alternative strategies to accelerate therapeutic discovery and combat drug resistance. One such approach is drug repurposing, which refers to identifying new therapeutic applications for drugs that have previously been approved for different indications [33,34,35,36]. A few examples of drugs repurposed for CRC include angiotensin receptor blockers used to treat hypertension; metformin for diabetes management; aspirin, which is an antiplatelet agent; and celecoxib, an anti-inflammatory drug [36,37,38,39,40,41,42]. In Saudi Arabia, Alzahrani et al., 2025 [43] reported the potential of propranolol, a beta blocker, in suppressing cell proliferation and induced apoptosis in CRC cell lines. Another study utilized pH-triggered ultra-elastic nanovesicles to deliver the NSAID celecoxib, which demonstrated pronounced efficacy in chemically induced tumorigenesis [44]. Collectively, these findings reinforce the potential of drug repurposing by proving the effect of non-oncology drugs to provide alternative solutions for CRC treatment.

Advancements in computational frameworks have refined drug repurposing strategies, facilitating high-fidelity predictions of therapeutic efficacy within complex biological systems. Consequently in silico screening has become a critical prerequisite for characterizing molecular interactions and prioritizing lead candidates for experimental in vitro validation [45]. This step results in a significant reduction in experimental costs. It is noteworthy that only one out of every 5000 drug candidates successfully reach the market [46]. Recent high-throughput genomic and proteomic studies have identified molecular targets associated with CRC. Recent studies [47,48,49] reported *CLSTN3*, *CLUH* and *SLC11A2* as novel targets that remain largely unexplored in the context of CRC therapeutics. However, the potential of these targets needs to be functionally validated to explore their potential in therapeutic development.

The CLU protein, originally discovered in a slime mold named *Dictyostelium discoideum*, is responsible for maintaining proper distribution of mitochondria within the cells [50,51]. In mammals, it is known as *CLUH* (Clustered Mitochondria Homolog) and performs similar function in regulating mitochondrial organization [50,52]. Loss of CLU function results in mitochondrial clustering around the nucleus and formation of multinucleated cells. A study [51] reported that *CLUH* regulated genes encoding mitochondrial proteins as well as those involved in ribosome biogenesis and translation. Specifically, CLUH binds to a subset of mRNAs that encode mitochondrial proteins [51]. Additionally, absence of CLUH causes significant reduction in key enzymes involved in catabolic energy-producing pathways in the mitochondria [53]. As a vital organelle, mitochondria play a pivotal role in energy metabolism, redox homeostasis and regulation of apoptosis. Hence, mitochondrial dysfunction is associated with a wide range of human cancers. It is reported that mutations in succinate dehydrogenase (*SDH*) have been reported in various cancers, including CRC [54,55].

Moreover, recent studies have shown that *CLUH* is a direct downstream target of *Msi2*. Overexpression of *CLUH* inhibits Msi2-induced mitochondrial dysfunction [56]. The RNA-binding protein Musashi-2 (*MSI2*) is confirmed to be overexpressed in CRC. MSI2 levels are elevated both in precancerous TAs (Tubular Adenoma) in the colonic mucosa and further during CRC progression [57]. Collectively, these findings highlight the critical role of *CLUH* in maintaining mitochondrial function and suggest that its dysregulation, potentially induced by MSI2 maybe associated with CRC.

The calsyntenin (CLSTN) family is a part of the superfamily of cell adhesion molecules. It comprises three members, namely *CLSTN1*, *CLSTN2* and *CLSTN3*. Our focus, CLSTN3 is a transmembrane protein predominantly localized in the postsynaptic membrane. Its extracellular region contains sex hormone-binding globulin and calmodulin repeats [58,59,60,61]. *CLSTN3* has not been studied in cancer; however, *CLSTN1* is reported to hold strong potential as a candidate biomarker for lung adenocarcinoma [62]. Interestingly, recent studies have shown the involvement of *CLSTN3* in regulating energy metabolism, obesity and related metabolic disorders [61,63,64]. CLSTN3 interacts with the amyloid precursor protein (APP) in the white adipose tissue (WAT) and disrupts mitochondrial function by promoting APP accumulation, which leads to obesity development [64]. Moreover, antisense-mediated suppression of APP in SW837 colon carcinoma cells significantly reduced their proliferation, colony formation in vitro and tumor growth in vivo. Also, knockdown of *APLP2*, a member of the APP family, resulted in reduced proliferation of the colon cancer cell line, Caco2 [65]. These findings support the investigation of *CLSTN3* as a potential candidate for CRC.

*SLC11A2* belongs to the solute carrier family 11, member 2 and functions as an important iron transporter [66,67]. The SLC11A2 protein mediates the absorption of ferrous ions from the intestinal lumen into the body through a transferrin-independent mechanism. This serves as the only pathway through which the gut absorbs dietary iron [67]. Ion channels are known to play a vital role in cellular communication and transport of materials and are linked to malignant transformation and cancer progression [68]. Several epidemiological studies have demonstrated a clear association between intake of dietary iron and an increased risk of cancer including CRC [69,70,71,72]. Furthermore, iron overload resulting from mutations in the HFE gene and/or excessive consumption of red meat rich in heme iron has been associated with higher susceptibility to CRC [72,73]. In addition, the expression of several genes associated with iron metabolism and transport is substantially altered in CRC tissues compared to normal colonic epithelium causing intratumoral iron accumulation [74,75,76]. Studies have also shown that excess iron may promote oxidative stress and activate the WNT signaling, thereby contributing to tumorigenesis [74,77]. Therefore, *SLC11A2* along with *CLUH* and *CLSTN3* might play a role in CRC development and progression and require detailed investigation to prove if they can serve as potential biomarkers for CRC treatment. This study aims to use drug repurposing strategies to identify existing therapeutic agents capable of targeting these novel proteins. Achieving this will not only accelerate therapeutic development but also help in overcoming current treatment limitations.

## 2. Materials and Methods

### 2.1. Ethical Approval

Ethical clearance for the study was granted by the Institutional Review Board (IRB) of Imam Abdulrahman bin Faisal University on 19th January 2025. The approval is valid for a period of one year under the IRB reference number: IRB-PGS-2025-13-0037.

### 2.2. Gene Selection

#### 2.2.1. Primary Screening to Identify New Potential Genetic Targets for CRC

Initial studies involved comprehensive screening through latest Genome-Wide Association Studies (GWASs) and meta-analysis studies on the PubMed database. Twenty-five genes were shortlisted based on their novelty as reported by recent genetic association studies [11,47,48,49].

#### 2.2.2. Literature Search for Confirming Novelty

Each shortlisted gene was cross-checked individually with the existing literature on PubMed, Google Scholar and Web of Science to confirm its novelty in association with CRC. The screening was narrowed down to nine candidate genes.

#### 2.2.3. Association with Other Cancers

The nine genes were further screened individually to check their association with other cancers. Seven out of nine genes were reported in other cancers (in vitro and in silico) based on the existing literature on PubMed.

#### 2.2.4. Final Selection of Potential Target Genes

Three candidate genes, namely *CLUH* (Clustered Mitochondrial Homolog), *CLSTN3* (Calsyntenin 3) and *SLC11A2* (Solute Carrier Family 11 Member 2), which have not been reported in CRC, were selected. Moreover, two of these genes, *CLUH* and *CLSTN3*, do not show association with other cancers.

#### 2.2.5. Independent Validation of Target Genes

Independent validation of CLUH, CLSTN3, and SLC11A2 was performed using publicly available databases (Table S1). Gene expression in colorectal cancer was analyzed using GEPIA2, which integrates TCGA and GTEx datasets, by comparing tumor and normal tissues in colon adenocarcinoma (COAD) and rectum adenocarcinoma (READ). Overall survival (OS) and disease-free survival (DFS) were evaluated using the Kaplan–Meier survival module in GEPIA2. Genomic alterations were assessed using cBioPortal across combined colorectal cancer cohorts. Protein expression was analyzed using the Clinical Proteomic Tumor Analysis Consortium (CPTAC) colon cancer dataset accessed through the UALCAN platform. Statistical significance was considered at *p* < 0.05.

### 2.3. Selection of Target Regions in CLUH, CLSTN3 and SLC11A2

#### Identification of Mutation-Enriched Exonic Regions

The Genomics Data Commons (GDC) Data Portal [78], maintained by the National Cancer Institute (NCI), was used to identify the somatic mutations in the selected genes. It provides access to large-scale cancer genomics datasets. Nearly 200–300 somatic mutations (Substitution, Frameshift, Insertion and Deletion) were reported in the portal for each selected gene. All the reported mutations were downloaded in an excel sheet and mutations with deleterious effect were filtered out. For each gene of interest, exon regions that had the highest mutations were selected and two highly mutated regions were chosen for further analysis, such as protein modelling and drug screening.

### 2.4. Mutation Score/Deleterious Effect Analysis Using In Silico Prediction Tools

#### 2.4.1. SIFT

The SIFT (Sorting Intolerant From Tolerant) algorithm was used to predict whether an amino acid substitution is likely to affect protein function. The prediction is based on sequence homology and the physicochemical similarity between the reference and substituted amino acids. For each substitution, SIFT provides a score and a qualitative prediction. The score ranges between 0 to 1 while the qualitative prediction indicates whether the substitution is tolerated or deleterious. SIFT score values closer to “0” have a higher potential of being deleterious. Substitutions with scores <0.05 were classified to be deleterious, while those ≥0.05 were considered tolerated.

#### 2.4.2. PolyPhen-2

PolyPhen-2 (Polymorphism Phenotyping v2) was also used to predict the impact of an amino acid substitution on the structure and function of a protein. The tool provided a score and a qualitative prediction indicated in Table S2. Both SIFT and PolyPhen-2 scores and quantitative predictions were obtained along with the somatic substitution mutations data downloaded from the GDC Data Portal [78].

#### 2.4.3. PANTHER

The PANTHER 19.0 (Protein ANalysis THrough Evolutionary Relationships) tool was employed to evaluate the potential functional impact of substitution mutations on protein function [79]. PANTHER utilizes Position-Specific Evolutionary Preservation (PSEP), which estimates the evolutionary preservation time (in millions of years) of an amino acid position by tracing it back through reconstructed ancestral protein sequences. Amino acid residues that have been conserved for longer evolutionary periods are more likely to be functionally important and substitutions at these sites are predicted to have deleterious effects. The PSEP score was further converted into a Probability of Deleteriousness (pDel) score, which was expressed both as a numerical probability and as a qualitative prediction. The thresholds used for interpretation are summarized in Table S3. For analysis, the PANTHER database [79] was accessed. Under the Genetic Variant Impact module, the protein sequence of the target gene was provided, along with the list of amino acid substitutions in the required format (e.g., *L46P*). The organism was specified as *Homo sapiens*, and predictions were obtained after submission.

#### 2.4.4. SNPs&GO

The SNPs&GO tool was also used to predict whether a given amino acid substitution is neutral or disease-related [80]. SNPs&GO combines protein sequence data, evolutionary information and functions based on Gene Ontology (GO) terms to estimate the potential impact of a substitution. A probability score >0.5 is disease and ≤0.5 is neutral. Protein sequences along with the corresponding amino acid substitutions were submitted to SNPs&GO platform and predictions were retrieved for further analysis.

#### 2.4.5. PhD-SNP

Predictor of human Deleterious Single Nucleotide Polymorphisms was also used to predict the effect of amino acid substitutions. It is based on a Support Vector Machine (SVM) that combines sequence information and evolutionary conservation to make predictions. The tool predicts whether a given substitution is disease-related or neutral and assigns a probability score, where values closer to “1” indicate a higher likelihood of being disease-associated, while values closer to “0” suggest neutrality. PhD-SNP scores can be obtained either directly from the PhD-SNP webserver (PhD-SNP: Predictor of human Deleterious Single-Nucleotide Polymorphisms) or through the SNPs&GO platform, which incorporates PhD-SNP as part of its prediction framework.

#### 2.4.6. CRAVAT

The Cancer-Related Analysis of Variants Toolkit was employed for genomic variant interpretation and functional annotation of mutations [81]. CRAVAT integrates multiple resources to provide information on the potential impact of variants, including their presence in the COSMIC database [82], which indicates the tissue type in which the mutation has been reported. For each non-silent variant, CRAVAT generates a VEST (Variant Effect Scoring Tool) *p*-value, which reflects the probability of the variant being pathogenic. Amino acid substitutions were submitted to the CRAVAT server in the required format, and the analysis was carried out using the VEST-4 prediction model along with GeneCard and PubMed annotations. The resulting pathogenicity scores and annotations were retrieved for further interpretation.

#### 2.4.7. Cumulative Significance

The Cumulative Score serves as a consensus-based filter to determine pathogenicity of mutation. Binary values were assigned to predictions from six independent algorithms (SIFT, PolyPhen-2, PhD-SNP, PANTHER, SNPs&GO and CRAVAT). A score of “1” was allocated for deleterious or high-impact predictions, while “0” represented neutral variants. The final score reflects the total number of tools reaching a consensus on a damaging nature of variant. This multi-tool validation ensures robust selection of deleterious variants for subsequent structural homology modelling and molecular docking analysis.

### 2.5. Protein Modelling and Structural Visualization

#### 2.5.1. Homology Modelling

Protein modelling was conducted using SWISS-MODEL (https://swissmodel.expasy.org/) [83] and PyMOL (Version 1, Schrödinger, LLC, New York, NY, USA) [84] to generate both wild-type and mutant structures of the selected gene. Initially, the protein FASTA sequence for the gene of interest was retrieved from the NCBI database and saved as a plain text file using Notepad. This sequence was then submitted to SWISS-MODEL by navigating to the Start Modelling option. The FASTA sequence was pasted into the interface, and a template search was performed, followed by the automated model building process. The resulting 3D structure was verified to ensure it corresponded to the correct gene and organism. The generated model was downloaded in PDB format and designated as the wild-type protein structure.

#### 2.5.2. Mutated Models

This PDB file was then opened in PyMOL for further structural analysis and mutagenesis. To create mutant models, PyMOL was launched and the wild-type PDB structure was loaded. Under the Display tab, the protein sequence was made visible and the background color was set to white for better contrast. From the displayed sequence, individual amino acids targeted for mutation were selected. The selected residue was visualized in stick format (via the “S” option), labeled (“L” option) and color-mapped using the C option in the menu by applying the rainbow spectrum. The mutagenesis wizard was then activated. Within the wizard, the target amino acid was substituted with the desired mutant residue. After previewing the rotamer, the mutation was applied and finalized. Confirmation was done by checking the updated residue in the sequence viewer. To save the mutant model, the modified structure was exported using the following sequence: File > Export Structure > Export Molecule. Under export options, the setting “Write header for every object” was selected. The file was saved in PDB format by changing the file type from PDBx/mmCIF (*.cif, *.cif.gz*) to PDB (.pdb, *.pdb.gz). This process was repeated to generate separate models for each amino acid substitution.

To ensure structural integrity and conformational accuracy, both experimental crystal structures and computationally predicted models were evaluated. The 3D atomic coordinates of the target protein (PDB ID: 9f6n.1.A) were retrieved from the Protein Data Bank. Structural analysis identified a co-crystallized Mn^2+^ ion in the active site, coordinated by key residues including Gln476, Met295 and Asp116. To prepare the structure for virtual screening, the bound ion was removed prior to molecular docking with known reference ligands. Post-docking protein–ligand interactions were analyzed using Discovery Studio and 2D interaction diagrams were generated (Figure S1).

#### 2.5.3. Structural Comparison of Wild and Mutated Models

To evaluate the structural impact of the prioritized missense mutations, the generated 3D models of the mutant proteins were superimposed onto their respective wild-type structures. The wild-type and mutant models for *CLUH*, *SLC11A2* and *CLSTN3* were imported into the Molecular Operating Environment (MOE) software [85]. A structural alignment was performed to superimpose the protein backbones and extract the Root Mean Square Deviation (RMSD) to know the structural deviation resulting from the mutation.

For a detailed analysis of local conformational changes, the superimposed structures were visualized using PyMOL. This tool was used to examine the specific amino acid substitutions and to visualize the resulting conformational changes, alterations in hydrogen bonding and shifts in packing density at the mutation sites.

### 2.6. Molecular Docking-Based Virtual Screening for Drug Repurposing

#### 2.6.1. Preparation of Ligand Library

All FDA-approved drugs up to 24 March 2025 were downloaded in .sdf format from the ZINC20 database [86] (University of California, San Francisco). These compounds were selected to ensure that only clinically approved drugs were included in the study. The ligands were processed using PyRx (Python, version 1.1 Prescription Virtual Screening Tool) [87], which was used for screening against both the wild-type protein and its mutant models. The Open Babel tool in PyRx was used to import 100 ligands per batch for analysis. To improve the quality of the structures, the three-dimensional geometry of each ligand was optimized by applying the *minimize all* function in PyRx. This energy minimization step removed unfavorable conformations and produced more stable ligand structures. After optimization, the ligands were converted into .pdbqt format using the *Convert all to Autodock Ligand* option in PyRx. This format ensures compatibility with AutoDock-based docking studies using PyRx.

#### 2.6.2. Drug Screening Against CLUH Protein

To evaluate the interaction of the ligands with CLUH wild-type protein, the PDB file was imported from the *Load Molecule* option in PyRx. The imported structure was converted into a macromolecule using the *AutoDock* tool in PyRx. Docking simulations were carried out through the Vina Wizard interface. The prepared ligands were selected and docking frame of the CLUH protein was adjusted to cover majority of the surface. The docking process was initiated by selecting the *Forward* option. PyRx then screened the drugs to generate binding affinity values and protein–ligand interaction models for further analysis. This process was repeated until all 1615 FDA-approved drugs were screened.

#### 2.6.3. Prioritization of Drugs

After completion of docking runs, the results were exported in CSV format. In addition, PDB files of ligand–protein complexes having the highest binding affinity were downloaded. The exported results were filtered in Microsoft Excel to identify drugs with the highest binding affinities. Top 15 candidate drugs were shortlisted. Out of these, three drugs were chosen for in vitro testing on colorectal cancer cell lines (HCT116 and HT29) and cell line from non-cancerous origin (HEK293). This selection was based on binding affinity, commercial availability and cost of the drug. This ensured that only suitable and accessible drug candidates were advanced for experimental validation.

#### 2.6.4. Extended Screening Against SLC11A2 and CLSTN3

The aim at this stage was to identify drug candidates that exhibited strong binding across all three target proteins (CLUH, CLSTN3 and SLC11A2) in both their wild-type and mutant forms. To achieve this, the top 150 drugs from the initial screening results having binding affinities of ≤–9.3 kcal/mol, were selected for further screening. The 150 selected drugs were docked against both wild-type and mutant models of SLC11A2, CLSTN3 and CLUH using PyRx. To identify the common hits/ligands across all proteins, results from each run were subjected to Venn diagram analysis using the Draw Venn Diagram tool. As a result, the selected list of drugs was narrowed down to 11 common drugs. Among these, only three drugs were selected for further in vitro investigation. This selection was based on commercial availability and cost of the drug. Antiviral drugs, compounds already used in CRC treatment and drugs available only in hospitals were excluded from the final selection.

#### 2.6.5. MD Simulation

A 100 ns molecular dynamics (MD) simulation was performed for the SLC11A2 mutant (Trp179Ser) in complex with Telmisartan using Desmond [version 4.1]. The analysis was performed with the OPLS-2005 forcefield, solvated using an explicit water model within an orthorhombic periodic boundary box with a 10 Å buffer. Following system preparation, energy minimization and relaxation, production runs were carried out in the NPT ensemble at 300 K and 1 bar for 100 ns. To quantify binding affinity, the molecular mechanics generalized Born surface area method was used using the VSGB implicit solvation model. Binding free energy ∆Gbind was calculated across the final 50 trajectory frames at one-frame intervals by summing individual energetic contributions, including van der Waals, electrostatic, hydrogen bonding, lipophilic and solvation components. This MD and free energy protocol validate the stability of the docked poses and confirms the therapeutic prioritization of Telmisartan.

### 2.7. In Vitro Validation Using MTT Assay

#### 2.7.1. Cell Lines and Culture Conditions

The human colorectal cancer cell lines HCT116 (accession no. CCL-247) and HT29 (accession no. HTB-38) along with non-cancerous origin cell line HEK293 (accession no. CRL-1573) were obtained from the American Type Culture Collection (ATCC, Manassas, VA, USA).

#### 2.7.2. Cell Culture Medium Preparation

Gibco DMEM (Dulbecco’s Modified Eagle Medium) with GlutaMAX™-I was supplemented with 50 mL fetal bovine serum (FBS), 5 mL of L-glutamine, penicillin–streptomycin (PenStrep) and non-essential amino acids (NEAA). All reagents are from Thermo Fisher Scientific, Waltham, MA, USA).

#### 2.7.3. Revival of HEK293, HCT116 and HT29 Cell Lines

Cell revival was carried out under sterile conditions using a LABCONCO Purifier Biological Safety Cabinet (Class II, Type A2, equipped with a HEPA filter). Before beginning the procedure, complete Gibco DMEM (Dulbecco’s Modified Eagle Medium) with GlutaMAX™-l, previously stored at 4 °C, was placed in a 37 °C water bath to warm to physiological temperature. Cryopreserved cell vials stored at −80 °C were thawed by immersion in the 37 °C water bath until most of the surrounding ice had melted.

The thawed cells were gently transferred into 5 mL of fresh DMEM in a sterile 15 mL centrifuge tube. Samples were centrifuged at 250× *g* (Eppendorf Centrifuge 5810R) for 5 min to pellet the cells. Following centrifugation, the supernatant was carefully aspirated and discarded. The resulting cell pellet was resuspended in 2 mL of fresh DMEM by gentle pipetting to ensure proper dispersion of the cells. In preparation for seeding, a 10 cm culture dish was supplemented with 10 mL of pre-warmed DMEM. The cell suspension was then added to the culture dish according to experimental requirements. The cells were observed under an inverted microscope (Nikon Eclipse TS100 Inverted Routine Microscope, Nikon Corporation, Tokyo, Japan) to confirm even distribution and initial viability. Cultures were incubated at 37 °C and 5% CO_2_ (Thermo Scientific Heracell 150i CO_2_ Incubator, Thermo Fisher Scientific, Waltham, MA, USA) in a controlled environment. Daily microscopic examinations were performed to monitor cell attachment, morphology and proliferation.

#### 2.7.4. Cell Seeding

Cell seeding was performed in 96-well plates. Prior to seeding, cells were washed with phosphate-buffered saline (PBS) and detached from the culture flask by trypsinization. To neutralize the trypsin, 5 mL of Dulbecco’s Modified Eagle Medium (DMEM) was added. The resulting cell suspension was transferred into a 15 mL centrifuge tube and centrifuged at 250× *g* for 5 min. The supernatant was discarded and the pellet was resuspended in 2 mL of fresh DMEM.

For cell counting, 10 µL of Trypan Blue dye (Thermo Invitrogen Trypan Blue Stain 0.4%) was mixed with 10 µL of the resuspended cells in a 1.5 mL centrifuge tube. The mixture was then loaded onto a cell counting slide and viable cell numbers were determined using the Invitrogen Countess II Automated Cell Counter.

For the MTT assay, cells were seeded at a density of 20,000 cells per well in a 96-well plate. The required volume of cell suspension was calculated using the following formula:

Cells needed (µL) = (Cells required/Cells counted) × 1000

The volume of fresh DMEM was then determined as

Fresh DMEM needed (µL) = Total volume (µL) − Cell suspension volume (µL)

The calculated volume of cell suspension was mixed with fresh DMEM in a solution basin and pipetted gently to ensure uniform distribution. A 100 µL quantity of the prepared suspension was then added into each well of the 96-well plate. Finally, the plates were incubated at 37 °C for 24 h under standard culture conditions before drug treatment.

#### 2.7.5. Drug Preparation and Treatment

Stock concentrations of the selected repurposing candidates were prepared by dissolving the compounds in DMSO. Venetoclax, Azilsartan medoxomil and Digoxin were prepared at stock concentrations of 20 mg/mL and 5 mg/mL. Telmisartan and Dutasteride were prepared at stock concentrations of 40 mg/mL and 5 mg/mL. The positive control, Cisplatin, was prepared at 0.25 mg/mL stock solution in PBS. Working solutions for cell treatment prior to the cytotoxicity assay were prepared by diluting the stock solutions in complete DMEM. To study the dose-dependent cytotoxicity and determine the half-maximal effective concentration (EC_50_), the cells were treated with four graded concentrations of each drug. The specific experimental concentrations used for each drug are detailed in Table S4. The experimental design included two controls: The non-treatment control (NTC) and the vehicle control (VC). For the NTC, cells were added with only fresh DMEM and for the VC, cells were treated with DMEM containing equivalent volume of DMSO to each concentration of the drug used. All drug treatments and controls were performed in experimental triplicates and repeated in four independent biological repeats (n = 4) for all cell lines (HCT116, HT29, HEK293).

#### 2.7.6. MTT Assay

Cell viability following drug treatment was evaluated using the MTT assay. After 48 h of treatment, the culture medium was removed from each well and the cells were washed with 90 µL of PBS. An initial absorbance reading was recorded at 570 nm using the Agilent BioTek Synergy Neo2 Hybrid Multi-Mode Microplate Reader. Representative images for each drug concentration were then captured using a microscope (Nikon Eclipse TS100 Inverted Routine Microscope) at 10× magnification. Following this, 10 µL of MTT (SIGMA) reagent (5 mg MTT in 1 mL PBS) was added to each well under dark conditions to prevent light-induced degradation. The plate was covered with aluminum foil and incubated at 37 °C for 3–4 h, allowing for the viable cells to reduce MTT into insoluble formazan crystals. After incubation, the solution was carefully removed. To solubilize the crystals, 100 µL of dimethyl sulfoxide (DMSO) was added to each well. The plate was placed on a thermomixer for approximately 10 min to ensure complete dissolution of the formazan crystals. Finally, the absorbance was measured at 570 nm using Agilent BioTek Synergy Neo2 Hybrid Multi-Mode Microplate Reader and the values were used to analyze the relative cell viability across different drug concentrations. Cell viability assay was performed 4 times for each drug and each cell line.

#### 2.7.7. Data Analysis

##### Calculation of Cell Viability

Following the measurement of absorbance, the raw OD values from the technical replicates were averaged from each treatment condition. Background correction was performed by subtracting the mean absorbance of the MTT blank wells (containing culture media and MTT reagent without cells) from the absorbance of all experimental wells. Cell viability % was calculated relative to the VC (DMSO-treated cells). This was done to normalize the cell viability against DMSO. The percentage cell viability for each dose was calculated using the following formula:

$$\mathrm{Cell}\;\mathrm{Viability}\;\left(\%\right)\;=\;\frac{{\mathrm{Mean}\;\mathrm{OD}}_{\mathrm{treatment}}-{\mathrm{Mean}\;\mathrm{OD}}_{\mathrm{blank}}}{{\mathrm{Mean}\;\mathrm{OD}}_{\mathrm{vehicle}\;\mathrm{control}}-{\mathrm{Mean}\;\mathrm{OD}}_{\mathrm{blank}}}\times 100$$

The final mean percentage cell viability and standard deviation (SD) were calculated from the independent biological repeats of each dose.

##### Statistical Analysis

Statistical analysis was performed using One-Way Analysis of Variance (ANOVA) with post hoc Tukey HSD Test Calculator with Scheffé, Bonferroni and Holm multiple comparison—input k, the number of treatments [88]. The post hoc analyses were employed to assess the statistical significance in two contexts. Firstly, to study the dose-dependent effects which involved comparing the viability at each drug concentration against the NTC. Secondly, to assess the differential selectivity by comparing the viability between the CRC cell lines (HCT116 and HT29) and non-cancerous origin cell line (HEK293) at the same dose concentrations. A *p*-value of <0.05 was considered statistically significant.

#### 2.7.8. Calculation of EC_50_ and EC_20_

EC_50_ and EC_20,_ represent half-maximal effective concentration and the concentration required to achieve 20% growth inhibition, respectively. All analyses were performed using GraphPad Prism version 10 (GraphPad Software, Boston, MA, USA). To calculate EC_50_, the experimental drug concentrations were first transformed to their logarithmic values. The corresponding cell viability data (Y-axis) was normalized, defining the smallest mean value as 0% and the largest mean value as 100%. The data was fit into a non-linear regression model using the equation: “log(agonist) vs. normalized response—Variable slope” (four-parameter logistic regression). The resulting EC_50_ was then used to calculate EC_20_ using GraphPad Software (Calculate ECanything from EC50).

### 2.8. Drug Treatment for Gene Expression Analysis of Telmisartan

#### 2.8.1. Cell Seeding

Cell seeding was performed in 6-well plate and a density of 1 × 10^6^ cells per well was used for HCT116, HEK293 and HT29. Initially, the harvested cell pellet was resuspended in 2 mL of fresh Dulbecco’s Modified Eagle Medium (DMEM) to prepare a uniform cell suspension. The cell mixture was gently pipetted to ensure homogeneity. To determine the viable cell count, 10 µL of Trypan Blue stain was added to 10 µL of the cell suspension in a sterile microtube. After mixing 10 µL of the mixture was loaded onto a disposable cell counting chamber slide (EVE™). The total viable cell count was measured using the Invitrogen Countess II Automated Cell Counter. The required volume of cell suspension was calculated using the following formula:

Cell suspension volume (µL) = (Cells required/Cells counted) × 1000

The volume of fresh DMEM to be added was calculated by subtracting the volume of cell suspension from the desired total volume. The calculated volumes of cell suspension and medium were mixed thoroughly in a sterile solution basin to ensure even distribution. Subsequently, 2 mL of the prepared cell suspension was seeded into each well of a sterile 6-well plate. The plate was then incubated at 37 °C and 5% CO_2_ (Heracell 150i CO_2_ incubator).

#### 2.8.2. Drug Treatment

To evaluate the differential expression of the target genes, HCT116, HT29 and HEK293 cell lines were treated with Telmisartan. After 24 h of seeding, the old DMEM was aspirated and cells were treated with Telmisartan at two experimentally determined concentrations: EC_50_ and EC_20_. The specific concentrations used for each cell line are detailed in Table S5. The Vehicle control group was treated with a volume of DMSO equivalent to the EC_50_ and EC_20_ dose of each cell line. The plates were incubated for 24 h post-treatment at 37 °C and 5% CO_2_. Following the incubation period, the cells were harvested immediately for total RNA extraction. The experiment was repeated three times to have three biological repeats.

#### 2.8.3. RNA Extraction

Total RNA was extracted from mammalian cultured cells using the GeneJET RNA Purification Kit (Thermo Scientific, Waltham, MA, USA), following the manufacturer’s protocol with minor optimizations. Prior to beginning the procedure, 20 μL of 2 M dithiothreitol (DTT) was added per 1 mL of lysis buffer to ensure RNA stability.

First, DMEM was first aspirated and discarded. Cells were rinsed once with PBS to eliminate residual medium, followed by PBS removal. To detach the cells, 200 μL of trypsin was added to each well and the plates were incubated at 37 °C for 3–5 min. Subsequently, 2 mL of PBS was added per well to neutralize trypsin and collect cells by pipetting. The cell suspension was transferred to a sterile microcentrifuge tube and centrifuged at 250× *g* for 5 min. The supernatant was discarded and the resulting cell pellet was either processed immediately or stored at −80 °C for later use. The pelleted cells were resuspended in 600 μL of lysis buffer supplemented with DTT and the suspension was vortexed for 10 s to ensure complete lysis. To this, 360 μL of 96–100% ethanol was added and the mixture was gently pipetted to mix. The lysate (up to 700 μL) was transferred into a GeneJET RNA purification column placed in a collection tube and centrifuged at ≥12,000× *g* for 1 min. The flow-through was discarded and the column was reinserted into the collection tube. This process was repeated until the entire lysate had passed through the column. The purification column was then placed in a fresh 2 mL collection tube. A volume of 700 μL of Wash Buffer I (pre-supplemented with ethanol) was added to the column, followed by centrifugation at ≥12,000× *g* for 1 min. After discarding the flow-through, 600 μL of Wash Buffer II (ethanol-supplemented) was added and the column was again centrifuged under the same conditions. A final wash was performed by adding 250 μL of Wash Buffer II and centrifuging at ≥12,000× *g* for 2 min to ensure complete removal of impurities. The RNA purification column was then transferred to a sterile RNase-free 1.5 mL microcentrifuge tube. For elution, 100 μL of nuclease-free water was added directly to the center of the membrane, allowed to stand at room temperature for 5 min and then centrifuged at ≥12,000× *g* for 1 min. The column was discarded and the purified RNA was either used immediately for downstream applications or stored at −80 °C for future use. Concentration and purity of the obtained RNA was measured using Thermo Scientific NanoDrop 8000 Spectrophotometer.

#### 2.8.4. cDNA Synthesis

First-strand cDNA was synthesized using the SuperScript™ VILO™ cDNA Synthesis Kit by Invitrogen—according to the following protocol: A master mix was prepared with the following components mentioned in Table S6. Following labelling, 10 µL of the RNA sample was added to the respective reaction tubes. Then, 10 µL of the prepared master mix was added to all tubes, including the negative control. The reactions were mixed thoroughly by vortexing. The cDNA synthesis was performed using the cycling conditions mentioned in Table S7. The cDNA was stored at −20 °C until further use.

#### 2.8.5. RT-PCR Primer Design

For expression studies, primers were designed to target the gene of interest using Primer designing tool from NCBI [89]. The mRNA sequence of the target genes (*CLUH*, *CLSTN3* and *SLC11A2)* and two housekeeping genes (*GAPDH* and *ACTB)* was first searched and downloaded in FASTA format from the NCBI database. This sequence was then used in an nBLAST search to confirm its reference genome (RefSeq) location. Primer design was done based on the following criteria: an annealing temperature between 60 and 63 °C, primer lengths of 20–30 base pairs, and amplicons between 250 and 350 base pairs were selected. The designed primers were checked using Primer-BLAST from NCBI. The gene’s FASTA sequence, along with the forward and reverse primer sequences, were entered into the tool. The Refseq mRNA was selected and the organism (*Homo sapiens*) was specified. The results were reviewed to ensure that the primers specifically targeted the correct gene and organism, with no off-target matches. The designed primers as indicated in Table S7 were then ordered from Integrated DNA Technologies (IDT) and prepared a working stock 10 mM concentration.

#### 2.8.6. Gene Expression Profiling

Quantitative real-time PCR (qRT-PCR) to assess the differential gene expression of *CLUH*, *CLSTN3*, *SLC11A2* (target genes) and *GAPDH* (reference gene) was performed using the Power SYBR™ Green PCR Master Mix (Applied Biosystems). The reaction mixtures were prepared in a final volume of 10 µL per well. The components of the master mix are shown in Table S9. For each primer pair and sample, 2.0 µL of cDNA was loaded into the reaction plate in triplicates to ensure reproducibility. Subsequently, 8.0 µL of a prepared reaction mix was added to each well, including the negative control. Following the addition of reagents, the reaction plate was sealed with an optical adhesive film and briefly centrifuged to eliminate air bubbles and ensure all components were collected at the bottom of the wells. The plate was then loaded into the Applied Biosystems 7500 Real-Time PCR System. The thermal cycling was performed using the standard “Comparative C_T_ (ΔΔ C_T_)” mode with the profile indicated in Table S10.

#### 2.8.7. Gene Expression Data Analysis

The raw Cycle Threshold (C_T_) values obtained from the qRT-PCR were exported for analysis. To ensure data accuracy, the C_T_ values from the technical triplicates were first averaged for each sample. The relative expression levels of *CLUH*, *SLC11A2* and *CLSTN3* were determined using the comparative C_T_ method (2^−ΔΔC^_T_). First, normalization was performed to obtain the Δ C_T_ for each sample using the formula (Δ C_T_ = C_T(target)_ − C_T(reference)_). Subsequently, the ΔΔ C_T_ was derived using the formula (ΔΔ C_T =_ Δ C_T(treated)_ − Δ C_T(VC)_). The relative fold change was the calculated using the formula 2^−ΔΔC^_T_ and converted to Log Fold Change (LogFC) to visualize the magnitude of upregulation or downregulation. This calculation was performed individually for each of the three independent biological replicates and the results were expressed as the mean LogFC ± standard deviation. Statistical significance of the differential expression across the cell lines in the two treatment groups was evaluated using One-Way Analysis of Variance (ANOVA) followed by post hoc multiple comparison tests Tukey’s HSD, Scheffé, Bonferroni and Holm. A *p*-value < 0.05 was considered statistically significant.

## 3. Results

### 3.1. Primary Screening to Identify Novel Potential Targets for CRC

**Figure 1:**
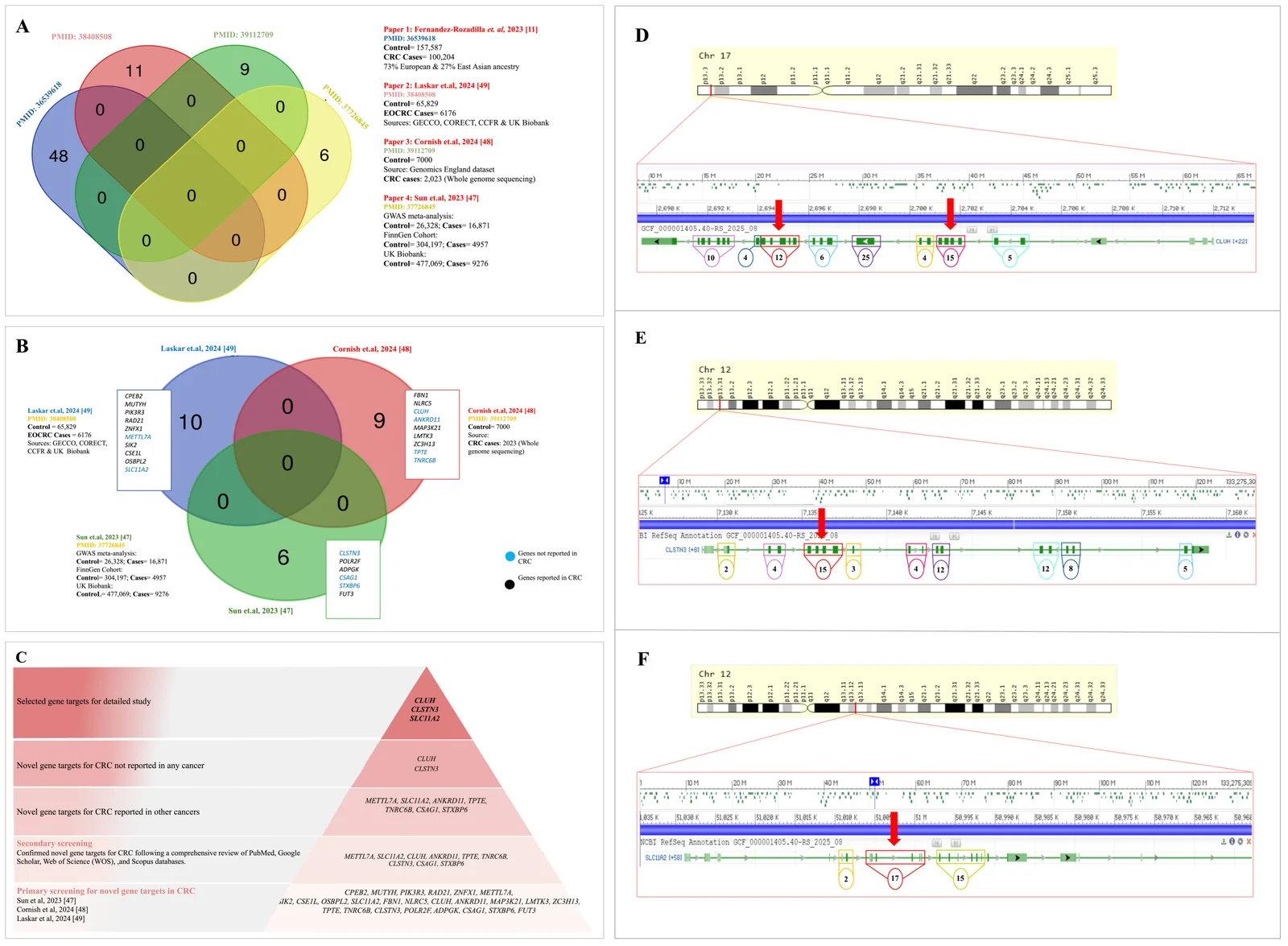
(**A**) Primary screening of four recent GWASs to identify new potential genetic targets for CRC [11,47,48,49]. (**B**) Newly reported genes in CRC based on [47,48,49]. Genes in blue color indicate novel genes associated with CRC and are not reported in CRC after literature validation. Genes in black color indicate genes that have already been reported in CRC. (**C**) Systematic Screening and Selection Strategy for Colorectal Cancer Genetic Targets. (**D**) Identification of Mutational Hotspots and Amplicon Selection for *CLUH* (Chromosome 17). (**E**) *CLSTN3* (Chromosome 12). (**F**) *SLC11A2* (Chromosome 17). The red arrow indicates regions with highest number of mutations selected for further analysis.

To identify the potential genetic targets associated with CRC, a primary screening was performed by investigating four recent large-scale Genome-Wide Association Studies (GWASs) as seen in Figure 1A. Each study included extensive case–control cohorts and represented diverse populations from various sources. A comparative analysis of the novel target genes reported in these studies was carried out using a Venn diagram (Figure 1A) to identify novel targets that were common across the four studies. However, the screening revealed no overlap of novel candidate genes. In total, the initial comparative analysis found 74 unique genetic targets, 48 from [11], 11 from [49], nine from [48] and six from [47]. This lack of overlap indicates a high degree of heterogeneity in the genetic associations reported by recent GWASw and suggests that the genetics of CRC are complex and influenced by the population, study design and analytical methods used. Despite the absence of common candidate genes, the primary screening forms a basis for further investigation of the newly proposed genes to confirm their association with CRC.

### 3.2. Literature Search for Confirming Novelty

The candidate targets identified by [47,48,49] during the initial screening were validated through a literature search to determine if these proposed targets had any previously reported association with CRC. The study by [11] was excluded from the study as it reported a large number of targets. The intersection of the three datasets is visualized a three-way Venn diagram (Figure 1B). This diagram shows the list of 25 unique genetic targets identified by the three studies and categorizes the proposed genetic targets from each study based on their association with CRC.

The literature search validated that out of the 25 genes, 16 were previously reported in CRC. This narrowed down the list of potential novel targets to nine genes for further investigation.

### 3.3. Association with Other Cancers

The nine genes identified in the previous section were further investigated for their association with eleven other major cancer types through a focused literature search. This step aimed at identifying targets that were highly specific to CRC. Table 1 summarizes these findings where a cross mark (**X**) in white and blue rows indicate in silico- and in vitro-validated association, respectively. Out of nine genes, seven have been linked to one or more cancer types, with *METTL7A* exhibiting the widest range of associations. Notably, *CLUH* and *CLSTN3* were not found to be associated with any of the cancer types. Their novelty to cancer in the existing literature suggests that *CLUH* and *CLSTN3* are the most promising and potential CRC-specific novel targets that require further analyses.

### 3.4. Final Selection of Genetic Targets for In Vitro Investigation

To conclude the systematic screening process, the novel genes identified in the previous sections were narrowed down to determine the most promising candidates for detailed in vitro investigation. The complete workflow, showing how the gene list was progressively refined from the initial broad screening to the final selection, is summarized in Figure 1C. The final selection included three genes: *CLUH*, *CLSTN3* and *SLC11A2*. Since *CLUH* and *CLSTN3* showed no reported association with any cancer type including CRC, they were prioritized due to their novelty profile. Our literature review indicated that *SLC11A2* was reported to be upregulated in ovarian and breast cancer. The overexpression of *SLC11A2* suggests a potential oncogenic role and provides a rationale for further investigation in CRC. Thus, *SLC11A2* was also included in the final shortlist.

### 3.5. Selection of Target Regions in CLUH, CLSTN3 and SLC11A2

To select target regions for further in silico and in vitro studies, somatic mutation data for *CLUH*, *CLSTN3* and *SLC11A2* was retrieved from the GDC Data Portal Homepage [78]. Analysis of variant distribution revealed distinct gene clustering of somatic mutations across the gene (Figure 1D–F). This guided the selection of target amplicons for further analysis.

For *CLUH*, multiple high-density mutational clusters were identified (Figure 1D). Although a specific exonic region consisted of 25 mutations, this region was excluded from the screening due to its high GC content and the presence of repetitive poly-GC sequences, which restricted the optimal primer design. Consequently, two regions, having 12 and 15 mutations each, were selected for further study. Although the region with 15 mutations was a high-priority target, it also contained poly GC stretches that pose a risk of sequencing erroneous reads. Therefore, the region containing 12 mutations was included as a complementary target to ensure robust and reliable genomic coverage. The selection for CLSTN3 (Figure 1E) and SLC11A2 (Figure 1F) was straightforward. The regions exhibiting the highest mutation frequencies, with 15 unique variants for *CLSTN3* and 17 unique variants for *SLC11A2*, were directly prioritized for primer design and further analysis.

### 3.6. In Silico Prediction of Deleterious Effects and Prioritization of Genetic Variants

A comprehensive in silico analysis was performed to predict the functional impact or deleteriousness of genetic variants identified in the target genes (*CLUH*, *CLSTN3* and *SLC11A2*). This analysis utilized six independent prediction tools, which included SIFT, PolyPhen-2, PANTHER, SNP&GO, PhD-SNP and CRAVAT. The scores from individual tools were combined to calculate a cumulative pathogenicity score (CPS). A score closer to 1.0 suggests a higher confidence that the variant is pathogenic. Variants were prioritized based on high CPS (≥0.8) and presence in large intestine tissue according to the COSMIC database. Genetic variant that met both conditions was selected for further analysis.

#### 3.6.1. Selection of CLUH Variants

**Figure 2:**
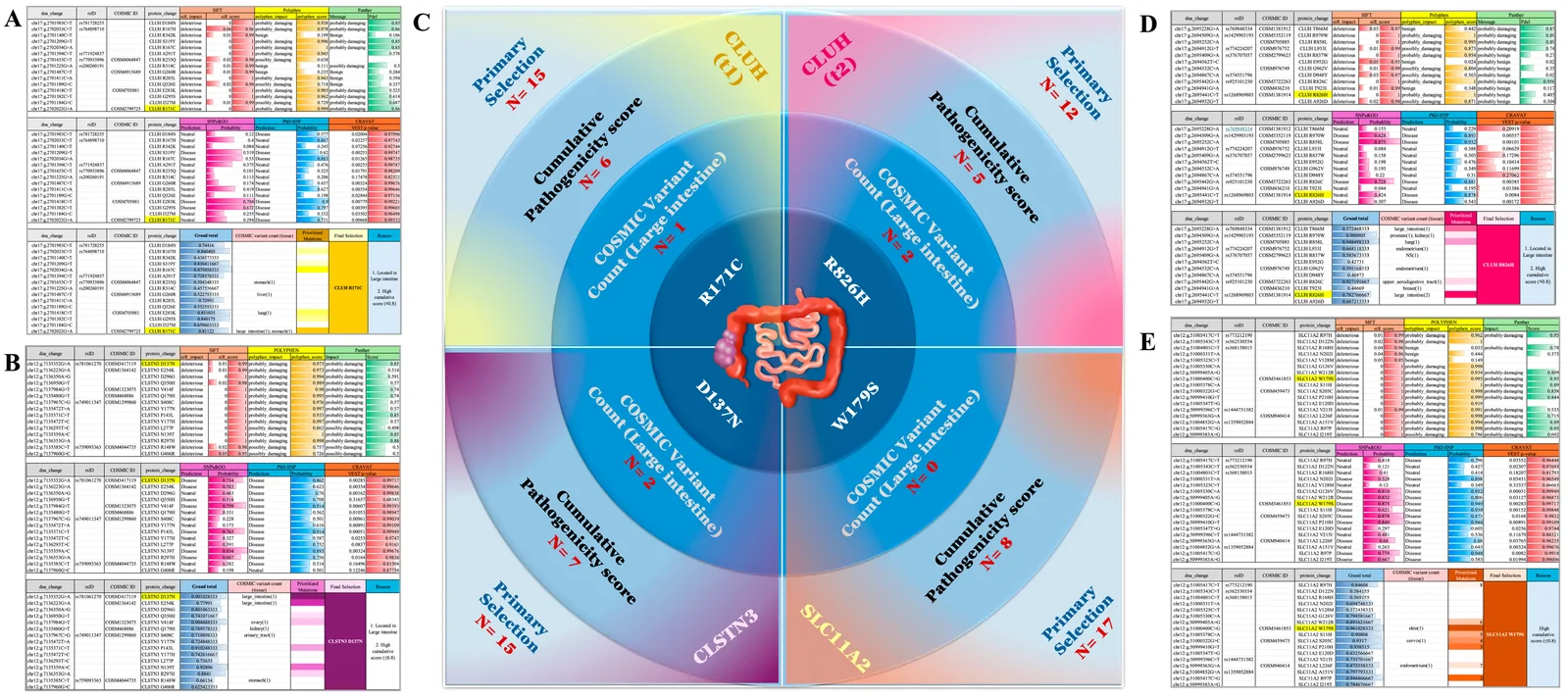
(**A**) In silico prediction of deleterious effects for CLUH variants Region 1 (CLUHt1). (**B**) In silico prediction of deleterious effects for *CLSTN3* variants. (**C**) Summary of in silico prediction and prioritization of genetic variants. (**D**) In silico prediction of deleterious effects for *CLUH* Region 2 variants (CLUHt2). (**E**) In silico prediction of deleterious effects for *SLC11A2* variants. Colour bars in (**A**,**B**,**D**,**E**) visualise the probability score for pathogenicity predicted by the various tools. The length and intensity of the bar correspond to the probability value.

The analysis of the *CLUH* gene was divided into two regions (Region 1 and 2) as shown in Figure 2A (Region 1) and Figure 2D (Region 2). In Region 1 (CLUHt1), the variant CLUH R171C was selected due to a high CPS of 0.811 and was reported in the large intestine, making it highly relevant to CRC. In Region 2 (CLUHt2), the variant CLUH R826H was prioritized. Although its CPS of 0.783 was slightly below general threshold, it was the only high scoring variant which was specifically reported twice in the large intestine. Other variants such as CLUH R858L had a higher CPS (0.948) but were found in unrelated tissues such as lung or prostate, making them less relevant to CRC.

#### 3.6.2. Selection of CLSTN3 and SLC11A2 Variants

The in silico prediction results for *CLSTN3* and *SLC11A2* genes are presented in Figure 2B,E, respectively. For the *CLSTN3* gene (Figure 2B), the variant D137N was identified as the most significant mutation due to its high CPS of 0.901 and was reported in the large intestine tissue, making it a clear candidate for selection.

For the *SLC11A2* gene (Figure 2E), the variant W179S was selected as this variant displayed the highest CPS of 0.9601 among all variants in the prioritized region. This specific region of the *SLC11A2* gene did not report any large intestine mutation in the COSMIC database. As a result, variant (W179S), with the highest CPS, was selected for further analysis.

The systematic in silico screening narrowed the list of potential genetic variants from each gene to four specific variants. A summary of the systematic selection strategy is shown in Figure 2C. It displays the transition from a broader selection pool to the final four candidate variants. Based on the combination of high CPS and relevance to the large intestine tissue, the variants CLUH R171C, CLUH R826H, CLSTN3 D137N and SLC11A2 W179S were prioritized for subsequent drug screening to identify therapeutic agents capable of modulating their deleterious effects for CRC treatment.

#### 3.6.3. Mutations Reported in the Large Intestine

The mutation screening identified 43 coding variants reported in large intestine from all exons of *CLUH*, *CLSTN3* and *SLC11A2* genes (Table S11). These mutations should be screened in CRC samples for their existence and association with development of CRC.

### 3.7. Protein Modelling and Structural Visualization

**Figure 3:**
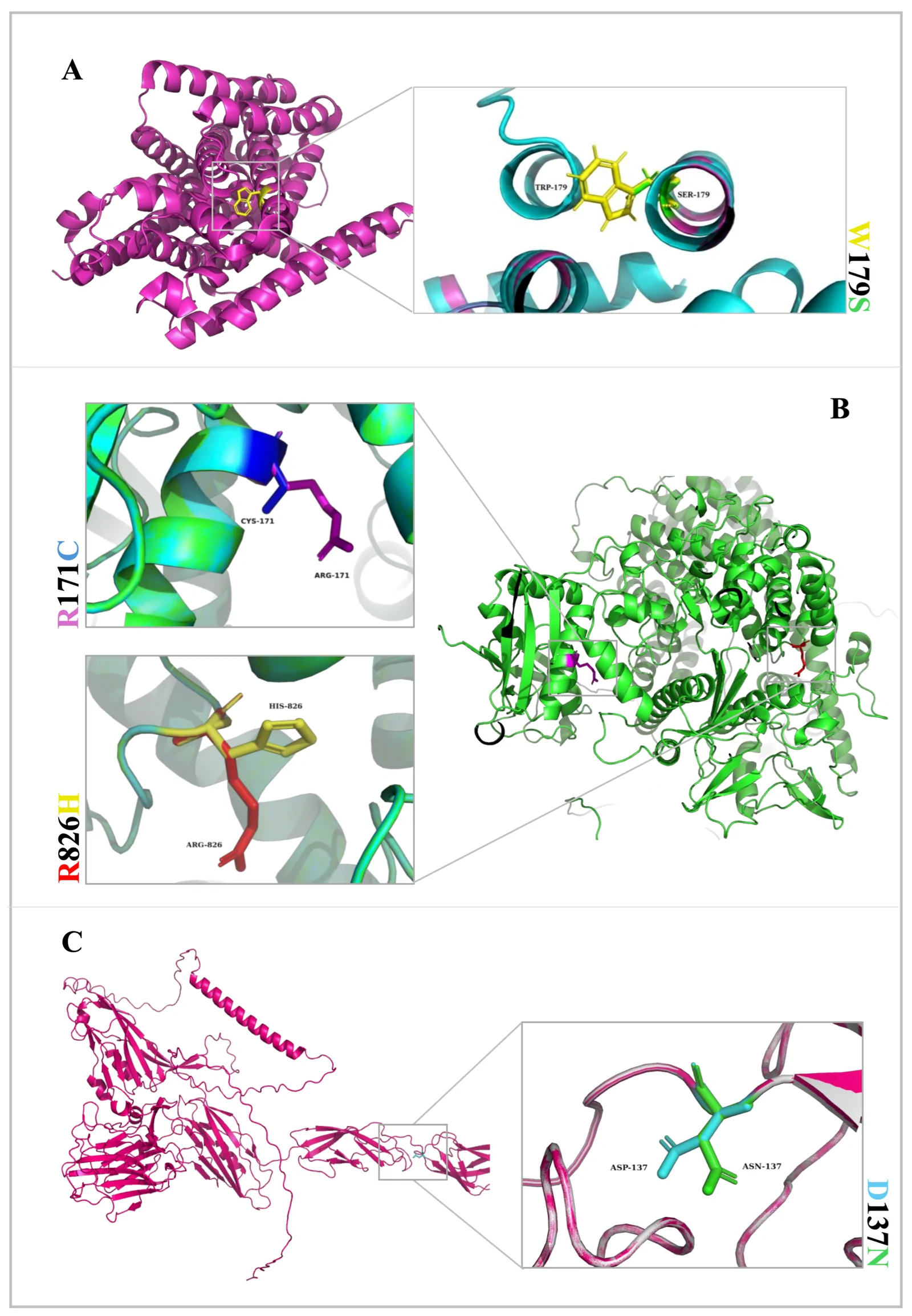
Comparative homology modelling and structural superimposition of wild and mutant proteins: (**A**) Superimposition of SLC11A2 models (wild type and mutant) illustrating substitution of tryptophan (W179—highlighted in yellow) with serine (S179—highlighted in green). (**B**) Superimposition of CLUH wild type (R171—highlighted in purple; R826 highlighted in red) and mutant form where arginine is substituted by cysteine and histidine, respectively (C171—indicated in blue; H826—highlighted in yellow). (**C**) Superimposition of CLSTN3 models illustrating substitution of aspartic acid (D137—indicated in cyan) with asparagine (N137—highlighted in green).

To further investigate the molecular basis of the predicted deleterious effects, three-dimensional (3D) protein models were constructed for both the wild-type and mutant forms of the selected targeted genes (*SLC11A2*, *CLUH* and *CLSTN3*) using SWISS-MODEL. The mutant models were superimposed onto their respective wild-type structures (Figure 3) using MOE and PyMOL to visualize the specific conformational changes and side-chain deviations at the mutation sites.

The structural impact of the amino acid substitution in SLC11A2 protein is shown in Figure 3A. The substitution of tryptophan with serine (Trp179Ser) results in a significant reduction in side-chain volume. The loss of the bulky aromatic ring of tryptophan and its replacement with the much smaller serine residue is likely to disrupt the local packing of the protein core. The structural alignment yielded a RMSD value of 0.073 Å. RMSD is a quantitative metric used to measure the average distance between the atoms of superimposed protein structures. RMSD was calculated to assess the magnitude of structural deviation, where a higher value indicates a significant conformational change in the mutant protein compared to the wild-type structure. This low RMSD indicates that the mutation does not cause substantial structural changes in the backbone; however, it introduces a significant local alteration in the packing density and hydrophobic interactions.

The two prioritized mutations in the CLUH protein can be visualized in Figure 3B. For CLUH R171C, arginine side-chain, which is long and positively charged, is replaced by a shorter, sulfur-containing cysteine. This alteration leads to a loss of positive charge and a reduction in side-chain length. Similarly, CLUH R826H involves the substitution of arginine with histidine. While both are basic residues, the geometric difference between the linear arginine and the ring-structured histidine introduces steric changes at the site. The calculated RMSD value for the CLUH mutant models was 0.001 Å, confirming that the overall fold is preserved but the physiochemical properties at these specific residues are significantly altered.

Finally, the structural analysis of the CLSTN3 protein is presented in Figure 3C. The amino acid substitution D137N involves the replacement of the negatively charged aspartic acid with the neutral, polar asparagine (Asp137Asn). While the size of the residues is similar, the loss of the negative charge alters the local electrostatic potential. The structural alignment showed an RMSD value of 0.003 Å. Collectively, these structural visualizations confirm that the deleterious nature of these mutations is not due to structural unfolding, but rather due to precise local deviations in charge, size and chemistry that likely compromise protein function or ligand binding.

To ensure structural integrity and conformational accuracy, both experimental crystal structures and computationally predicted models were evaluated. The 3D atomic coordinates of the target protein (PDB ID: 9f6n.1.A) were retrieved from the Protein Data Bank. Structural analysis identified a co-crystallized Mn^2+^ ion in the active site, coordinated by key residues including Gln476, Met295 and Asp116. To prepare the structure for virtual screening, the bound ion was removed prior to molecular docking with known reference ligands. Post-docking protein–ligand interactions were analyzed using Discovery Studio and 2D interaction diagrams were generated ( Figure S1 ).

### 3.8. Molecular Docking-Based Virtual Screening for Drug Repurposing

**Figure 4:**
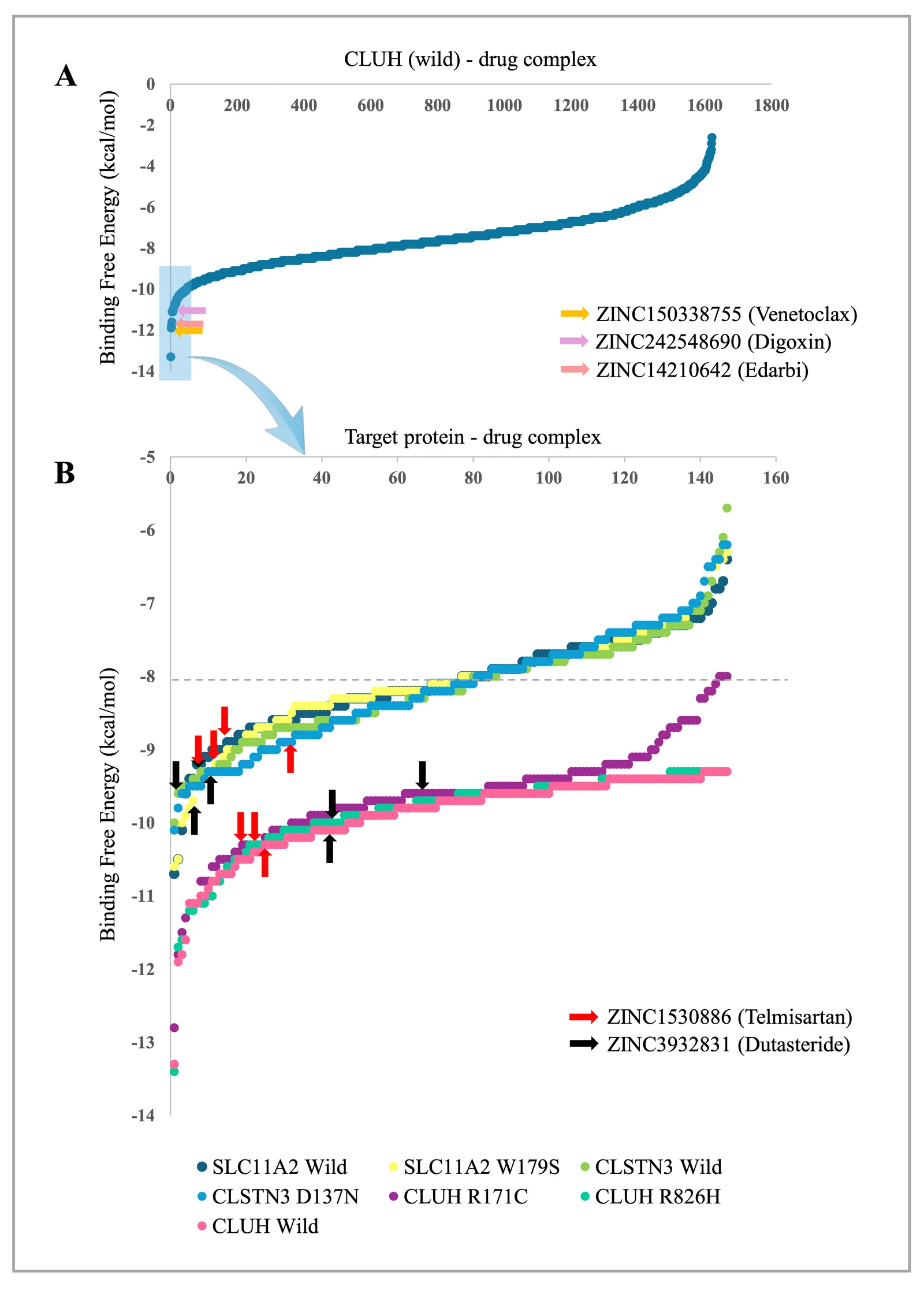
Sequential high-throughput virtual screening and candidate selection. The plots illustrate the binding free energy profiles (ΔG) obtained from molecular docking. (**A**) Primary Screening: Docking of 1615 FDA-approved drugs against the wild CLUH protein. The orange, purple and pink arrows represent Venetoclax (ZINC150338755), Digoxin (ZINC242548690) and Azilsartan medoxomil (ZINC14210642), which were selected for immediate testing. The blue shaded region indicates the top 150 hits. (**B**) Secondary Screening: Evaluation of the top 150 drugs against the complete panel of wild-type and mutant targets. The dashed line indicates the selection threshold of ΔG ≤ −8.0 kcal/mol. The red and black arrows indicate the specific binding affinity points for the final selected drug candidates from secondary screening: Telmisartan (ZINC1530886) and Dutasteride (ZINC3932831).

The next aim was to identify potential therapeutic agents capable of targeting the prioritized proteins which include the wild types and their mutant forms. A multistage high-throughput virtual screening was conducted using a library of 1615 FDA-approved drugs retrieved from the ZINC20 database. The overall workflow for screening and sequential selection is illustrated in Figure 4.

The initial screening (Figure 4A) involved docking the complete library of 1615 drugs against the CLUH wild-type protein using PyRx. This primary step filtered the library based on binding affinity. The three top drug candidates, which were selected for further in vitro testing, were Venetoclax (ZINC150338755), Digoxin (ZINC242548690) and Azilsartan medoxomil (ZINC14210642). Venetoclax exhibited the highest binding affinity (−11.9 kcal/mol) and is used for the treatment of leukemia. Digoxin, indicated for heart failure, closely followed Venetoclax with a binding affinity of −11.8 kcal/mol followed by Azilsartan medoxomil, an anti-hypertensive drug displayed a binding energy of −10.6 kcal/mol. These drugs were prioritized due to their exceptional binding affinities and availability. Subsequently, top 150 drugs from the initial screening were subjected to a secondary screening against the complete panel of targets proteins (Figure 4B), which included the wild and mutant forms of CLUH, CLSTN3 and SLC11A2 for identifying common hits capable of targeting all selected variants with a high affinity. Furthermore, a threshold of ΔG ≤ −8.0 kcal/mol was applied across all targets for final filtering.

The common hits identified after the secondary screening and applying the cut-off are summarized in Table 2. This step identified 10 common drugs that consistently met the binding threshold against all targets. The final selection from this screening included two candidate drugs for repurposing, which are the Telmisartan (ZINC1530886) and Dutasteride/Avodart (ZINC3932831) (Figure 4B). The selection was based on consistent low binding affinity (ΔG ≤ −8.0 kcal/mol), safe clinical profiles, easy availability, cost-effective and suitable for long-term administration. Digoxin was already included during the initial screening. The remining drugs were excluded due to various limitations in terms of availability, cost, requirement for IV administration or because they were already in use for CRC treatment (Table 2). The specific positions of the five selected drugs are indicated by arrows (Figure 4B) showing where the binding affinity of the drug lies for each respective protein. Venetoclax and Azilsartan medoxomil from the initial step have a high binding affinity with the other targets; however, Telmisartan and Dutasteride are superior.

### 3.9. Chemical Structures of Selected Candidates

Following the rigorous virtual screening and prioritization process, the 2D chemical structures of the five final drug candidates were retrieved to visualize their structural diversity (Figure S2). The panel displays the scaffolds of Venetoclax (Figure S2A), Telmisartan (Figure S2B), Dutasteride (Figure S2C), Digoxin (Figure S2D) and Azilsartan medoxomil (Figure S2E). These compounds exhibit distinct pharmacological pharmacophores: Venetoclax possesses a complex structure designed to inhibit BCL-2; Telmisartan and Azilsartan medoxomil feature biphenyl tetrazole rings characteristic of angiotensin receptor blockers; Dutasteride displays a steroid-like 4-azasteroid structure; and Digoxin is a cardiac glycoside composed of a sugar and steroid nucleus. This structural heterogeneity suggests that the selected candidates utilize diverse mechanisms to interact with the target proteins.

### 3.10. Molecular Interaction and Binding Mode Analysis

**Figure 5:**
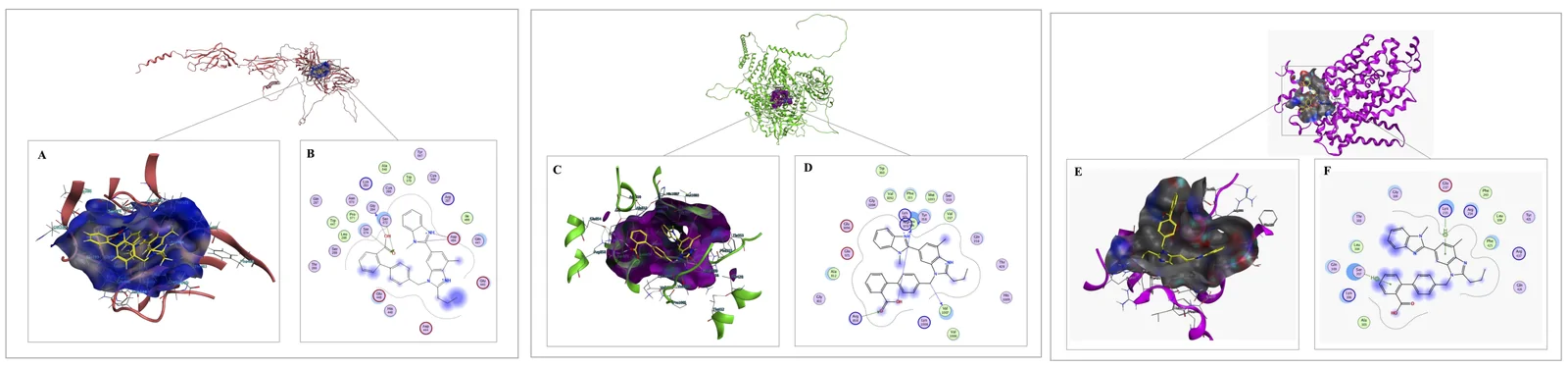
Molecular interaction and binding orientation of Telmisartan complexes with representative target proteins. (**A**,**C**,**E**) 3D surface models showing the binding pockets and orientation of Telmisartan (coloured yellow) within (**A**) CLSTN3, (**C**) CLUH, and (**E**) SLC11A2, highlighting optimal geometric and physiochemical complementarity. (**B**,**D**,**F**) 2D interaction maps corresponding to the complexes of Telmisartan with (**B**) CLSTN3, (**D**) CLUH, and (**F**) SLC11A2, respectively. The binding is stabilized by a network of non-covalent interactions, including hydrogen bonds (represented by green dashed lines) and hydrophobic interactions, validating the high binding free energies observed in virtual screening.

To elucidate the structural basis of the high binding affinities observed in virtual screening, molecular interaction analyses were performed using MOE for representative drug–protein complexes. The complexes of Telmisartan with CLSTN3, CLUH and SLC11A2 are represented in Figure 5. The 3D surface models (Figure 5A,C,D) and 2D interaction maps (Figure 5B,D,F) reveal that binding is stabilized by a network of non-covalent interactions. Telmisartan fits into the binding pockets of all three proteins through hydrogen and hydrophobic bonding. Thus, Telmisartan possesses optimal geometric and physiochemical complementarity to the respective targets. This validates the high binding free energies obtained in the virtual screening.

### 3.11. MD Simulation and Binding Stability of the SLC11A2 (Trp179Ser)–Telmisartan Complex

**Figure 6:**
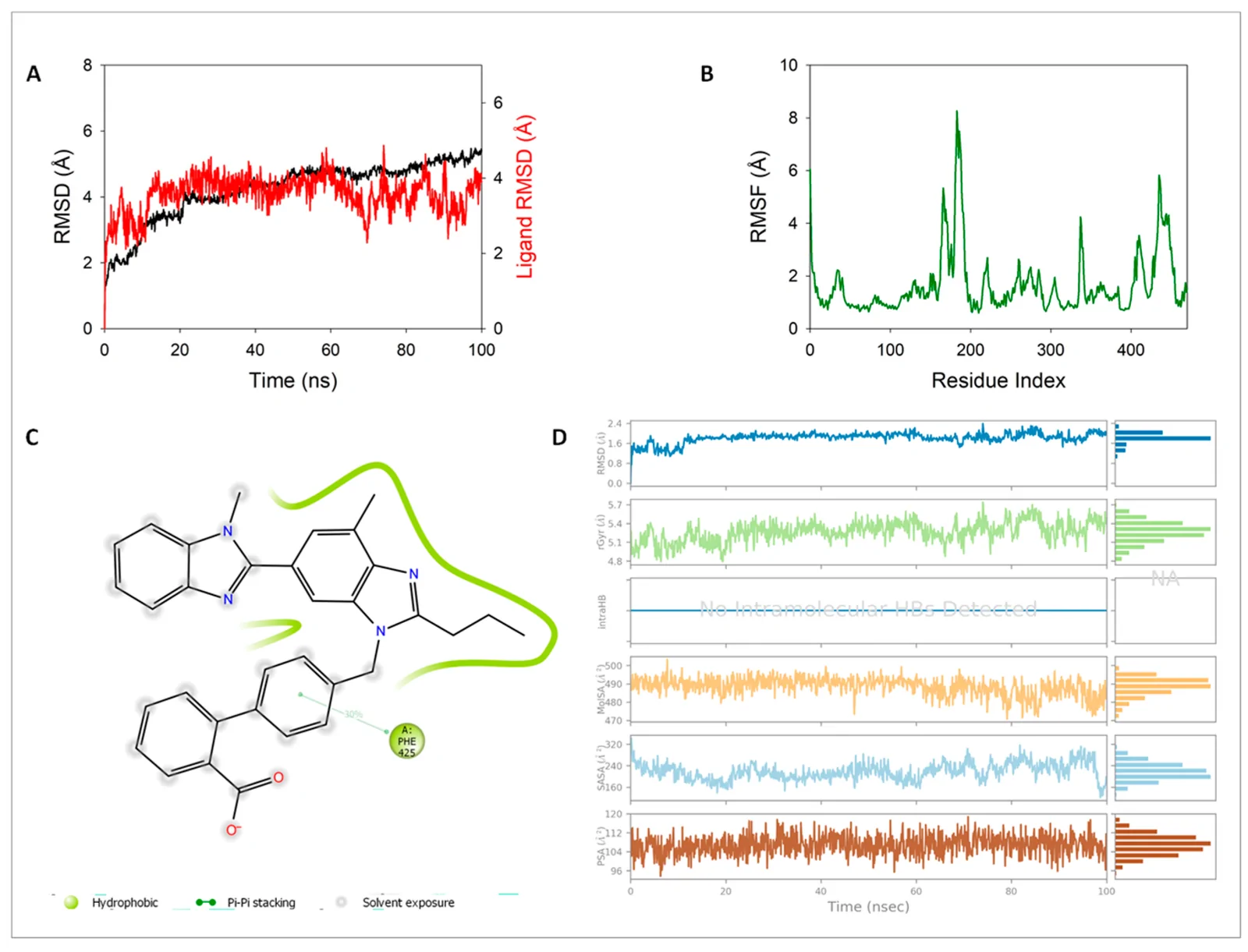
Molecular dynamics (MD) simulation analysis of the SLC11A2 (Trp179Ser) mutant in complex with Telmisartan over a 100 ns trajectory. (**A**) Root mean square deviation (RMSD) plot of the protein backbone and ligand (red) indicating structural convergence and binding stability. (**B**) Root mean square fluctuation (RMSF) of the protein residues, demonstrating stable local conformations across the backbone. (**C**) Protein–ligand contact interactions categorized by various bonds highlighting dominant interactions with key active site residues such as PHE 425. (**D**) Ligand property profiles confirm consistent conformational stability and structural compactness of Telmisartan in the binding pocket.

A 100 ns molecular dynamics (MD) simulation was performed to identify the docking reliability and provide dynamic validation for target engagement for the SLC11A2 mutant (Trp179Ser) in complex with Telmisartan. The root mean square deviation (RMSD) analysis of the protein backbone clearly revealed the stable convergence and acceptable range of structural fluctuation throughout the 100 ns production run for the SLC11A2 mutant (Trp179Ser)–Telmisartan complex. The ligand RMSD showed that Telmisartan achieved stable accommodation within the binding cavity of SLC11A2, with minor internal fluctuations but not leading towards ligand diffusion. Root mean square fluctuation (RMSF) data confirmed the residue flexibility remained stable around the active binding region. SLC11A2 mutant (Trp179Ser)–Telmisartan complex contact analysis revealed that the binding conformation is stabilized due to hydrophobic interactions with PHE 425 acting as a prominent residue maintaining hydrophobic for a major fraction of the simulation time (Figure 6). Analysis of ligand properties confirmed that Telmisartan preserves optimal geometric and physicochemical complementarity under physiological thermal fluctuations. These comprehensive MD simulation metrics effectively validated the virtual screening outcomes and confirm binding stability and provide severe biophysical justification for prioritizing Telmisartan in colorectal cancer treatment.

### 3.12. In Vitro Cytotoxicity Assessment (MTT Assay)

**Figure 7:**
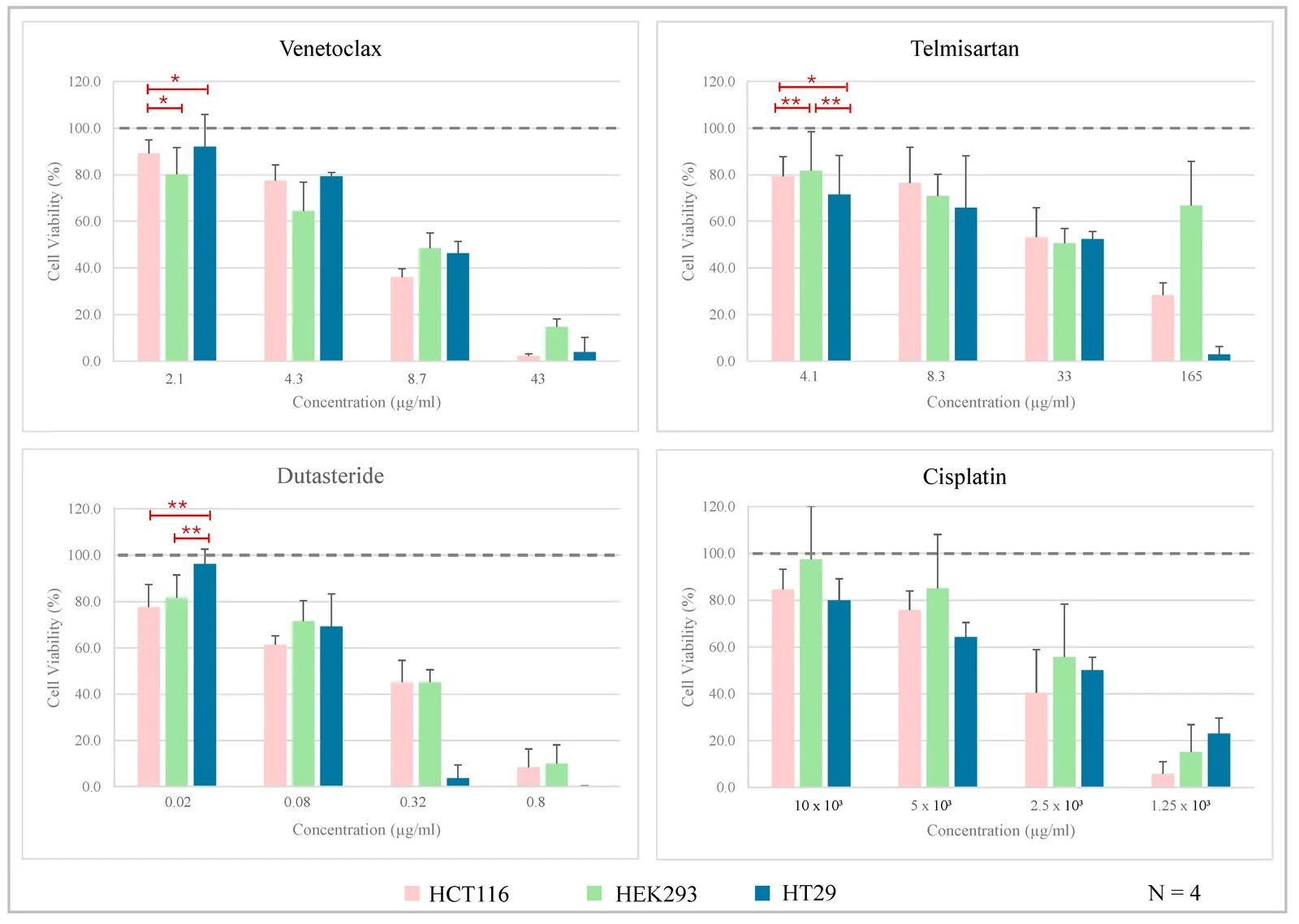
In vitro cytotoxic effects and comparative analysis (N = 4) of Venetoclax, Telmisartan, Dutasteride and Cisplatin (positive control). The colored bars represent mean ± SD of the corresponding dose; (*) and (**) indicates (*p* < 0.05) and (*p* < 0.01), respectively. Dashed line at 100% represents the non-treatment control (NTC) threshold. Statistical analysis for dose dependent cytotoxicity is shown in Table 3.

To experimentally validate the findings from the in silico screening, the cytotoxic efficacy of the selected drug candidates was evaluated using the MTT assay. The assay was performed on two colorectal cancer cell lines (HCT116 and HT29) and one non-cancerous human cell line (HEK293) to assess both potency and selectivity. Cisplatin was employed as a positive control to validate the assay sensitivity. Cytotoxicity assessment was performed on all the five prioritized drug candidates (Venetoclax, Telmisartan, Dutasteride, Digoxin and Azilsartan medoxomil) from the virtual screening analysis. The five candidate drugs and the positive control were tested across four different concentrations. The selection of treatment concentrations was optimized for each drug. Venetoclax, Telmisartan and Dutasteride demonstrated significant inhibitory effects. The dose-dependent cell viability profile is illustrated in Figure 7.

**Table 3:** Statistical analysis of dose-dependent cytotoxicity.

Cell Line	Drugs	Doses			
		Dose 1 vs. NTC	Dose 2 vs. NTC	Dose 3 vs. NTC	Dose 4 vs. NTC
HCT116	Venetoclax	-	*p* = **0.0010053	*p* = **0.0010053	*p* = **0.0010053
	Telmisartan	-	*p* = *0.0296905	*p* = **0.0010053	*p* = **0.0010053
	Dutasteride	*p* = *0.0234647	*p* = **0.0010053	*p* = **0.0010053	*p* = **0.0010053
	Cisplatin	-	*p* = *0.0280803	*p* = **0.0010053	*p* = **0.0010053
HEK293	Venetoclax	-	*p* = **0.0025094	*p* = **0.0010053	*p* = **0.0010053
	Telmisartan	-	*p* = *0.0322214	*p* = **0.0010053	*p* = **0.0129722
	Dutasteride	*p* = *0.0221076	*p* = **0.0010053	*p* = **0.0010053	*p* = **0.0010053
	Cisplatin	-	-	*p* = *0.0269426	*p* = **0.0010053
HT29	Venetoclax	-	*p* = *0.0161162	*p* = **0.0010053	*p* = **0.0010053
	Telmisartan	*p* = *0.0423806	*p* = *0.0124711	*p* = **0.0010053	*p* = **0.0010053
	Dutasteride	-	*p* = **0.0031826	*p* = **0.0010053	*p* = **0.0010053
	Cisplatin	*p* = *0.0201139	*p* = **0.0010053	*p* = **0.0010053	*p* = **0.0010053

NTC: Non-treatment control; -: Insignificant; * *p *< 0.05; ** *p *< 0.01.

In Venetoclax treatment, a sharp, dose-dependent reduction in cell viability was observed across all cell lines. Statistical analysis (Table 3) confirmed that Venetoclax resulted in a highly significant reduction (*p* < 0.01) in cell viability compared to the non-treatment control (NTC) at Dose 2 (4.3 µg/mL), 3 (8.7 µg/mL) and 4 (43 µg/mL) in HCT116 and HEK293 cell lines. HT29 also shared a similar effect at Dose 3 and 4 (*p* < 0.01). Telmisartan treatment also resulted in a clear, dose-dependent decrease in cell viability across both cancer cell lines. A statistically significant reduction in cell viability was observed at Dose 3 (33 µg/mL) and Dose 4 (165 µg/mL) (Table 3). However, the decrease was more gradual compared to a rapid reduction observed with Venetoclax. Most importantly, Dose 4 (165 µg/mL) of Telmisartan in HCT116 and HT29 gives a more significant reduction in cell viability (*p* < 0.01) compared to HEK293 (*p* < 0.05), highlighting its potential as an anti-cancer agent that might be less toxic to healthy cells at the lowest dose. Dutasteride demonstrated consistent cytotoxic effects and statistical evaluation indicated significant reduction in viability (*p* < 0.01) across Dose 2 (0.08 µg/mL), Dose 3 (0.32 µg/mL) and Dose 4 (0.8 µg/mL) in both cancer cell lines (Table 3). This validates its potential as a repurposed therapeutic agent. Cisplatin, served as a positive control and gave an expected significant cytotoxic response (*p* < 0.01), which validated the experimental conditions.

A crucial criterion for drug repurposing is its ability to target cancer cells without inducing severe toxicity in healthy tissues. To evaluate this, a comparative analysis of cell viability was performed between the CRC cell lines (HCT116 and HT29) and the non-cancerous cell line (HEK293) followed by post hoc tests (Tukey HSD and Holm) as seen in Figure 6. This study revealed that Telmisartan has more significant statistical evidence of selective toxicity. At the highest dose (165 µg/mL), the reduction in cell viability of cancer cell lines (HCT116: *p* < 0.01; HT29: *p* < 0.01) was highly significant compared to HEK293 (*p* > 0.01) cell line. This selective nature of the drug at this concentration served as an ideal profile for repurposed as an anti-CRC agent. Venetoclax also shows significant difference in cytotoxicity (at the highest dose 43 µg/mL) between cancer cell lines (HCT116, HT29) and HEK293 cell line (*p* < 0.05); Dutasteride also demonstrates significant difference in HEK293 vs. HT29 (*p* < 0.01) at the highest dose, but no significant difference was observed between HCT116 and HEK293. All the other doses of the three drugs did not show any significant difference in the pairwise analysis; however, they are consistent in reducing the cell viability and statistically significant compared to the NTC (Table 3). Furthermore, treatment with Digoxin resulted in a dose-dependent reduction of cell viability in HT29 but not HCT116 (Figure S4). Conversely, Azilsartan medoxomil moderately affected HCT116 but not HT29 (Figure S4). Therefore, both Digoxin and Azilsartan medoxomil were not selected for further investigation. In conclusion, this study identified Telmisartan as the most promising compound for further investigation as a selective anti-CRC therapy. Venetoclax also demonstrates a selective nature; however, Telmisartan shows more prominent statistical significance. Dutasteride is less suitable due to its comparatively less selective nature.

### 3.13. Differential Gene Expression Analysis

Real-Time PCR was performed to investigate whether the observed cytotoxicity of Telmisartan was mediated through the modulation of the identified genetic targets. The expression levels of *SLC11A2*, *CLUH* and *CLSTN3* were measured in HCT116, HT29 and HEK293 cell lines after being treated with Telmisartan at EC_50_ (T50) and EC_20_ (T20) concentrations. The log fold change of *SLC11A2* and *CLUH* relative to the NTC is illustrated in Figure 7. The *CLSTN3* gene did not give consistent results in RT-PCR, making it difficult to analyze the effect of treatment on this gene.

**Figure 8:**
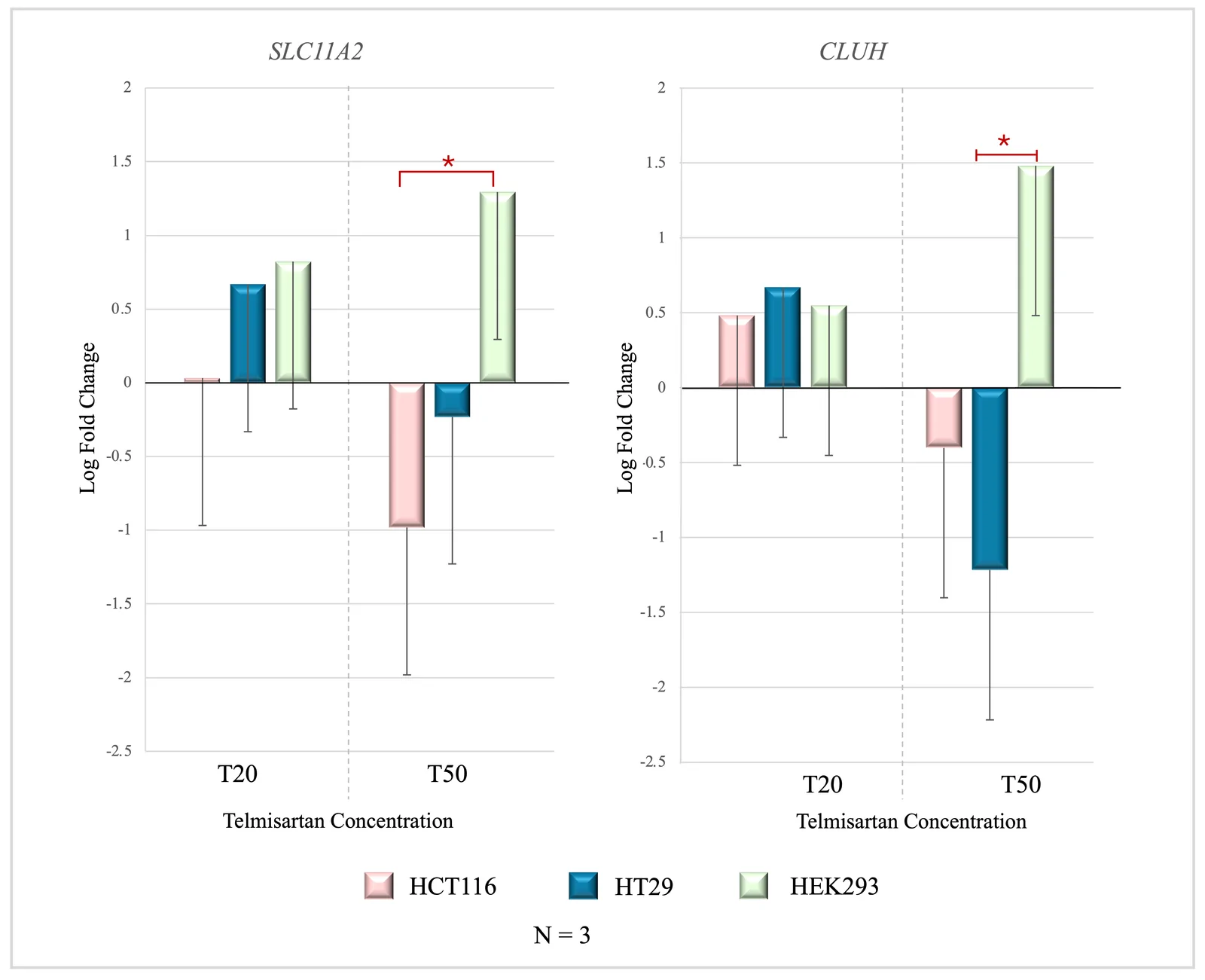
Differential gene expression of *SLC11A2* and *CLUH* (N = 3) following Telmisartan treatment at EC_50_ (T50) and EC_20_ (T20); the zero line indicates NTC threshold. A positive log fold change indicates upregulation (gene induction) and a negative log fold change indicates downregulation (gene repression). * Significant difference at *p* < 0.05.

*SLC11A2* has been reported to be upregulated in multiple cancers such as breast and ovarian cancer [67]. Telmisartan treatment induced a significant dose-dependent downregulation of this gene (Figure 8: Left Panel) in HCT116 and HT29. At the lower concentration (T20), no inhibitory effect was observed. However, at T50, a significant downregulation of the expression of *SLC11A2* was observed in both CRC cell lines (HCT116 and HT29). Moreover, the gene remained upregulated in the non-cancerous origin cell line (HEK293). The pairwise statistical analysis confirmed a significant difference (*p* < 0.05) in the expression levels of *SLC11A2* post treatment (T50) in HCT116 and HEK293 (Figure 8: Left Panel). This further confirms the selective nature of Telmisartan in downregulating the expression of *SLC11A2* in CRC cell lines. The high dose of Telmisartan induced a significant differential effect. The CRC cell lines (HCT116 and HT29) switched from mild induction (at T20) to significant repression of *SLC11A2*. However, the normal cell line (HEK293) revealed a mild upregulation (Figure 8: Left Panel).

The expression trend of *CLUH* (Figure 8: Right panel) followed a similar pattern with *SLC11A2* upon treatment with Telmisartan. The prognostic role of *CLUH* in CRC remains unknown in the current literature, making it difficult to assert definitive conclusions if its downregulation can contribute to anti-proliferative effects and tumor suppression. However, the gene expression data of *CLUH* confirmed that Telmisartan exerts a selective biological impact on CRC cells compared to non-cancer cells. At the T50 dose, Telmisartan induced a significant downregulation in both CRC cell lines (HCT116 and HT29) whereas the HEK293 cells exhibited a strong upregulation (Figure 8: Right panel). The observed differential response is statistically significant (in HCT116 vs. HEK293, *p* < 0.05), correlates with the cytotoxicity analysis and confirms the selective nature of Telmisartan. Overall, the differential gene expression analysis revealed the anti-cancer activity of Telmisartan involves targeted suppression of *SLC11A2* and *CLUH*. It also demonstrates desirable selectivity in downregulating the gene expression in CRC cells while maintaining normal or upregulated levels in non-cancerous cells. These observations have further validated the selection of Telmisartan as a potential repurposed drug for CRC treatment.

## 4. Discussion

Colorectal cancer remains as the second leading cause of cancer-associated mortality worldwide [90], yet the molecular mechanisms underlying CRC have not been completely understood [91]. This highlights the dire need for precise and novel genetic targets and therapeutic strategies. Therefore, the objective of our study was to identify novel molecular markers of CRC and screen for commercially available drugs that can modulate the expression of the selected genes as a part of the drug repurposing strategy. This study employed a comprehensive and multi-disciplinary approach that will help contribute to bridging the gap between large-scale GWASs and clinical application. We systematically screened recent multi-omics and GWAS articles and identified *CLUH*, *CLSTN3* and *SLC11A2* as new targets that might be associated with CRC [47,48,49]. This was followed by further identification of the mutation rich area within the gene from currently available information at the genomics cancer portal [78]. We identified that many mutations in these genes were reported in the large intestine. This was followed by in silico structural modelling and investigating the deleteriousness of the reported mutations. Experimentally testing all FDA-approved drugs is time-consuming and resource-intensive; therefore, computational screening to prioritize high-potential candidates is critical prior to in vitro validation [45]. High-throughput virtual screening of 1615 FDA-approved drugs against the targeted proteins resulted in identifying drugs with high binding affinity to these targets. The prioritized drugs were experimentally validated, and Telmisartan was prioritized as the best candidate to be repurposed for CRC treatment. Our observation is in line with earlier studies on repurposing Telmisartan for various cancers [92,93,94]. In addition to being selective against the tested cancer cells, Telmisartan also resulted in the downregulation of *SLC11A2* and *CLUH* expressions in colorectal cancer cell lines (HCT116 and HT29).

Precision medicine is a futuristic approach that has begun to achieve milestones in the field of oncology, especially in the effort to overcome CRC. The rapid development in genomics and bioinformatics has opened significant opportunities to analyze the detailed genetic profiles of individuals [95]. This allows for the precise identification of specific mutations present in the cancer cells [96]. In the context of CRC, genomic profiling facilitates the identification of common mutations in genes like *KRAS*, *BRAF*, *PIK3CA* and *TP53* [95,97,98,99,100]. These mutations play a critical role in cell signaling pathways that are involved in cell differentiation, proliferation and apoptosis [101]. Interpatient heterogeneity was observed in all mutations in patients with CRC. Therefore, by identifying the unique genomic profile of each patient, precision medicine can be designed and directed to target specific molecular pathways involved in the development and progression of CRC [95,102,103]. This approach allows for a more effective and targeted treatment along with potentially reduced side effects compared to conventional therapies [95,104]. However, these advancements still face limitations due to the resistance mechanisms and the complex heterogeneity of the disease [105,106]. Hence, there is an evident need for a wider range of molecular markers to further optimize the efficacy of targeted therapy and obtain the required outcome for patients. Tang et al. [107] emphasized that identifying new genetic targets that correlate with patient prognosis is essential in developing more specific drugs and improving the prognosis of patients with metastatic CRC (mCRC). Our study made efforts to contribute to this framework of expanding the therapeutic landscape by identifying *CLUH*, *CLSTN3* and *SLC11A2* as potentially associated novel targets. While genetic drivers like *KRAS* and *BRAF* are well-established in CRC, our in silico screening revealed these three potential genes were unexplored in CRC. Widely recognized targets are often subjected to resistance mechanisms; hence, novel genes like *CLUH*, *CLSTN3* and *SLC11A2* can offer alterative in CRC therapeutics. Our study observed the upregulation of *SLC11A2* in negative control, which is consistent with the confirmed upregulation in ovarian and breast cancer [67].

CLUH is an RNA-binding protein (RBP) that regulates the biogenesis and distribution of mitochondria within the cells [50,52]. Specifically, CLUH binds to a subset of mRNAs that encode mitochondrial proteins [51]. RBPs are closely associated with CRC progression and modulate critical tumorigenic processes such as cell proliferation and EMT [108,109]. Absence of CLUH causes significant reduction in key enzymes involved in catabolic energy-producing pathways in the mitochondria [53]. As a vital organelle, mitochondria play a pivotal role in energy metabolism, redox homeostasis and regulation of apoptosis. In cancer, tumor cells rely on robust mitochondrial function not only for energy production but also to regulate apoptosis and endure metabolic stress. This phenomenon is known as the Warburg effect. Hence, mitochondrial dysfunction is associated with a wide range of human cancers. It is reported that mutations in succinate dehydrogenase (SDH) have been reported in various cancers, including CRC [54,55]. Moreover, recent studies have shown that *CLUH* is a direct downstream target of *Msi2*. Overexpression of *CLUH* inhibits Msi2-induced mitochondrial dysfunction [56]. The RNA-binding protein Musashi-2 (MSI2) is confirmed to be overexpressed in CRC [57]. Collectively, these findings highlight the critical role of CLUH in maintaining mitochondrial function and suggest that its dysregulation, potentially induced by MSI2 maybe associated with CRC. However, our findings found that Telmisartan-induced downregulation of CLUH led to significant cancer cell death. Further detailed studies are required to confirm the state of *CLUH* in CRC. The in silico screening for mutations within *CLUH* gene identified about eighteen substitution and five frameshift mutations that were reported in the large intestine by the COSMIC database.

CLSTN3 (Calsyntenin-3) is a transmembrane protein predominantly localized in the post synaptic membrane. It is one of the three members of the Calsyntenin family which belongs to the cadherin superfamily [58,59,60,61,109]. Members of the cadherin superfamily such as E-cadherin and N-cadherin are associated with CRC [110]. *CLSTN3* has not been studied in cancer in current literature. However, The Human Protein Atlas has identified *CLSTN3* as a prognostic marker in breast invasive carcinoma and pancreatic adenocarcinoma [111,112]. CLSTN1 has been reported as a strong potential biomarker for lung adenocarcinoma [62]. Another study found that targeting a spliced isoform of *CLSTN1* was able to reverse ESRP1-induced invasion and metastasis of gastric cancer cells [109]. ESRP1, an RNA-binding protein, is reported to be overexpressed in CRC [108]. Furthermore, splice isoform switching of *CLSTN1* is a critical driver of EMT in breast cancer [113]. Interestingly, recent studies have shown that *CLSTN3* is involved in regulating energy metabolism, obesity and related metabolic disorders. CLSTN3 interacts with the amyloid precursor protein (APP) in the white adipose tissue (WAT) and disrupts mitochondrial function by promoting APP accumulation, which leads to obesity development [64]. Moreover, antisense-mediated suppression of APP in SW837 colon carcinoma cells significantly reduced their proliferation, colony formation in vitro and tumor growth in vivo. Also, knockdown of APLP2, a member of the APP family, resulted in reduced proliferation of the colon cancer cell line, Caco2 [65]. In our study, the in silico screening for investigating the deleteriousness of mutations reported within *CLSTN3* identified about twenty mutations that were reported in the large intestine by the COSMIC database.

SLC11A2 belongs to the solute carrier family 11, member 2 and functions as an important iron transporter [66,67]. The SLC11A2 protein mediates the absorption of ferrous ions from the intestinal lumen into the body through a transferrin-independent mechanism. This serves as the only pathway through which the gut absorbs dietary iron [67]. Iron addiction is a well-established concept in oncology. Ion channels are known to play a vital role in cellular communication and transport of materials and are linked to malignant transformation and cancer progression [68]. Studies have also shown that excess iron may promote oxidative stress and activate the WNT signaling, thereby contributing to tumorigenesis [74,77]. Several epidemiological studies have demonstrated a clear association between intake of dietary iron and an increased risk of cancer including CRC [69,70,71,72]. Furthermore, iron overload resulting from mutations in the *HFE* gene and/or excessive consumption of red meat rich in heme iron has been associated with higher susceptibility to CRC [72,73]. In addition, the expression of several genes associated with iron metabolism and transport is substantially altered in CRC tissues compared to normal colonic epithelium, causing intratumoral iron accumulation [74,75,76]. We included *SLC11A2* based on its novelty reported in the GWAS by Lasker et al. [49]. However, further literature review revealed that *DMT1* (*SLC11A2*) is linked to CRC via JAK-STAT3 signaling [114], which supports our bioinformatics results. Our study finding revealed that Telmisartan treated cancer cell lines (HCT116 and HT29) observed with downregulation of *SLC11A2* and reduction in CRC cell viability. Elaborative studies are needed to ensure the actual impact of Telmisartan on cancer cell lines (HCT116 and HT29). A study [115] demonstrated that Angiotensin II stimulation significantly increases the expression of iron transporters including SLC11A2. Since Telmisartan is an Angiotensin Receptor Blocker (ARB), this suggests that its treatment effectively neutralizes this signaling pathway. This study strongly aligns with our findings of Telmisartan-induced downregulation of *SLC11A2* in CRC cells. The observed differences in the viability between HCT116 and HT29 cell lines are because of their distinct natural biological characteristics and genetic landscapes. The HCT116 cell line is categorized as microsatellite instability (MSI) positive, exhibits the CpG island methylator phenotype (CIMP+), and lacks the chromosomal instability (CIN) pathway. Genetically, HCT116 harbors a *KRAS* mutation (G13D) and a *PIK3CA* mutation (H1047R), while maintaining wild-type status for *BRAF*, *PTEN*, and *TP53* [116]. In contrast, the HT29 cell line is microsatellite stable (MSS), demonstrates both CIMP+ and CIN+ characteristics, and features a distinct mutational profile including a *BRAF* mutation (V600E), a *PIK3CA* mutation (P449T), and a *TP53* mutation (R273H), while remaining wild type for *KRAS* and *PTEN*. These divergent genetic landscapes, specifically the presence of *KRAS* versus *BRAF* mutations and the difference in microsatellite and chromosomal instability pathways, likely contribute to the variations in baseline drug sensitivity and cell viability observed across our study. These differences are related with baseline sensitivity and are common in cancer research. As illustrated in Figure 7 despite variations in the magnitude of gene expression changes, Telmisartan consistently demonstrates a selective and significant effect of downregulation on the novel targets *SLC11A2* and *CLUH* in both colorectal cancer cell lines (HCT116 and HT29) at the T50 concentration. Both CRC cell lines showed a shift from mild induction at T20 to significant repression at T50 in the *SLC11A2* analysis. Also, Telmisartan induces significant downregulation in both HCT116 and HT29 at T50 in the *CLUH* analysis, which was not in line with the strong upregulation observed in the non-cancerous cell line. Collectively, these findings and genetic landscapes [116] suggest that while cell-line-specific variability exists, the targeted suppression of *SLC11A2* and *CLUH* represents a consistent, influence for the anti-proliferative effects of Telmisartan across CRC models.

Drug repurposing has emerged as a beneficial approach offering shorter time frame, established safety and cost effectiveness [33]. It has been reported that the costs can be 160 million times lower than traditional drug development. The majority of the cost is saved in safety testing, molecular characterization, safety profiling and initial marketing [36,117,118]. Such repurposed drugs targeting novel markers in combination with existing cancer treatments can prove to be a potent strategy to combat drug resistance and improve survival rates [119]. Our study prioritized drug candidates not only based on cytotoxicity but also on how suitable they are for clinical use, safety profiles and cost effectiveness.

One of the final candidate drugs was Venetoclax, which demonstrated significant potency against CRC cell lines. It is a well-established selective inhibitor of BCL2, which is a key anti-apoptotic protein often overexpressed in cancers that prevent cell death. Venetoclax is widely used in the targeted treatment of Chronic Lymphocytic Leukemia (CLL) and Acute Myeloid Leukemia [120]. Venetoclax is currently under clinical trial for ER-positive breast cancer [121] and small cell lung cancer [122]. These findings boost our selection of Venetoclax as a potential drug to be repurposed for CRC. BCL2 is frequently overexpressed in CRC and is correlated with resistance to standard chemotherapeutic agents such as 5-fluorouracil [123,124,125]. Our study identified that Venetoclax has a strong binding affinity against CLUH (−11.9 kcal/mol), in addition to showing significant cytotoxicity against CRC cells. Dutasteride functions as an inhibitor of 5-alpha reductase, which is used to primarily treat Benign Prostate Hyperplasia (BPH). It prevents the conversion of testosterone into the more active androgen dihydrotestosterone (DHT) [126]. Furthermore, the REDUCE (Reduction by Dutasteride of Prostate Cancer Events) trial proved that Dutasteride significantly lowers the risk of prostate cancer in men at a higher risk [127]. In our study, Dutasteride was demonstrated to have a high binding affinity across all selected targets (CLUH, CLSTN3, SLC11A2) and a potential inhibitor of CRC cell viability. This finding is supported by strong evidence reporting that the expression of Androgen Receptor (AR) was linked to tumorigenesis and immune invasion in CRC [128]. Collectively, these findings validate dutasteride as a promising candidate to be repurposed for CRC treatment.

Finally, we investigated Telmisartan, which is a classical Angiotensin II Receptor Blocker (ARB) primarily used for hypertension [129]. The literature supports that it also has anti-cancer effects mediated through mechanisms different from the typical renin–angiotensin system [130]. A recent study reported that Telmisartan has significant anti-proliferative effects on CRC cell lines in a dose-dependent manner [131,132]. This finding aligns with our study, which not only identified the potential cytotoxicity of Telmisartan but also its selectiveness in targeting cancer cells and being less toxic to the normal cells. This was proven both during the MTT assay as well as during the gene expression of *SLC11A2* and *CLUH*. It selectively downregulated both the genes only in HCT116 and HT29 while maintaining upregulated levels in HEK293. Moreover, Telmisartan has a well-established safety profile with excellent long-term tolerability [129]. While HEK293 cells are a widely accepted and robust model for assessing the baseline safety and systemic toxicity of therapeutic compounds, the use of non-malignant human colonic epithelial cells would provide a more tissue-specific assessment of selective efficacy. Consequently, the analysis of normal colonic epithelial models as a crucial next step for further validation of the safety profile of Telmisartan and therapeutic potential in future pre-clinical studies. The observed transcriptional changes in *SLC11A2* and *CLUH* were not validated at the protein level, which is considered as a limitation of the current study. Future studies are needed to assess the protein expression of these targets using Western blot or other proteomic approaches to further confirm the mechanism of action of Telmisartan in CRC and normal cell lines.

In summary, the in silico predictions, in vitro cytotoxicity assessment and differential gene expression analysis positioned Telmisartan as the most suitable candidate for drug repurposing in CRC. This is due to its outstanding ability to downregulate the identified targets *SLC11A2* and *CLUH* in CRC cell lines (HCT116 and HT29) while maintaining favorable levels in non-cancerous origin cells (HEK293). It addresses a critical need in current oncological therapies, which is the need for efficacy without inducing harmful toxicity.

## 5. Conclusions

The rising global burden of colorectal cancer (CRC) and the limitations of current standard-of-care therapeutics in terms of resistance mechanisms and severe systemic toxicity necessitate a shift towards precision medicine and safer therapeutic alternatives. This study successfully employed a comprehensive and multi-disciplinary framework involving in silico screening followed by in vitro validation to identify novel genetic markers and potential repurposing therapeutic agents. This research prioritized *CLUH*, *CLSTN3* and *SLC11A2* as potentially novel genetic targets in colorectal cancer from the recent GWASs. Exons with highest number of somatic mutations were targeted for molecular informatics analysis. In silico prediction of mutations with deleterious effects further prioritized specific variants within these genes based on their high cumulative pathogenicity score and presence in the large intestine according to the COSMIC database. Our study identified drugs with the potential to be repurposed for CRC. Homology modelling of the target proteins followed by molecular docking based virtual screening of all FDA approved drugs identified Venetoclax, Dutasteride and Telmisartan with the lowest binding energy and reflected positively on the in vitro cytotoxicity assessment. Additionally, 100 ns molecular dynamics simulations validated the dynamic stability and structural integrity of the SLC11A2 mutant–Telmisartan complex throughout the production run. Telmisartan was positioned as the most suitable among the candidates for drug repurposing in CRC. This is due to its widespread availability, established safety profile for chronic use and cost effectiveness suggests the prioritization of Telmisartan for further pre-clinical validation as an adjuvant in CRC treatment regimens. This further validates the MTT results where Telmisartan exhibited a significant difference in cytotoxicity between the CRC cell lines and HEK293. While *SLC11A2* is known to be upregulated in CRC, our study identified Telmisartan as a potential repurposed drug that significantly downregulates the expression of *SLC11A2* in CRC cell lines. It addresses a critical need in current oncological therapies, which is the need for efficacy without inducing harmful toxicity. Its widespread availability established safety profile for chronic use and cost effectiveness strongly suggests the prioritization of Telmisartan for further pre-clinical validation as an adjuvant in CRC treatment regimens. Future advanced studies using patient samples will confirm the involvement of these genes in CRC. Telmisartan was prioritized as an excellent candidate to be repurposed for CRC treatment; however, further confirmatory studies are required to better understand the effect of Telmisartan.

## Figures and Tables

**Table 1:** Association with other cancers.

	Ovarian Cancer	Prostate Cancer	Melanoma	Lung Cancer	Non-Small Lung Cancer	Breast Cancer	Renal Cancer	Hepatocellular Carcinoma	Thyroid Cancer	ESCC	HNSCC	Gastric Cancer
***METTL7A***				**X**		**X**	**X**	**X**				
			**X**						**X**			
***SLC11A2***												
	**X**					**X**						
***CLUH***												
											
***ANKRD11***												
	**X**					**X**						
***TPTE***												
		**X**	**X**		**X**							
***TNRC6B***		**X**		**X**								
										**X**	**X**	**X**
***CLSTN3***												
											
***CSAG1***			**X**									**X**
											
***STXBP6***												
				**X**	**X**	**X**						

**Table 2:** Selection and exclusion of common drug candidates from secondary screening with binding affinity ≤ −8.0 kcal/mol.

ZINC ID	Drug Generic/Compound Name	FDA Indication	Status	Reason for Selection/Exclusion
ZINC1530886	Telmisartan	Hypertension	✓	High binding affinity across all targets, Easy availability, Safe and cost friendly
ZINC3932831	Dutasteride	Benign Prostate Hyperplasia	✓	High binding affinity across all targets, Easy availability, Safe and cost friendly
ZINC164528615	Glecaprevir	Hepatitis C	✗	Antiviral; restricted availability and high cost
ZINC6716957	Nilotinib	Chronic Myeloid Leukaemia	✗	Already repurposed for CRC
ZINC3978005	Dihydroergotamine	Migraine	✗	IV injection; restricted availability
ZINC52955754	Ergotamine	Migraine	✗	IV injection; restricted availability
ZINC150338819	Ledipasvir	Hepatitis C	✗	Antiviral; restricted availability and high cost
ZINC12503187	Conivaptan	Hyponatermia	✗	IV injection; restricted availability
ZINC242548690	Digoxin	Atrial fibrillation (or) Heart failure	✓	High binding affinity across all targets, Easy availability
ZINC1612996	Irinotecan	Colorectal Cancer	✗	Already used to treat CRC

✓: Selected; ✗: Excluded.

## Data Availability

The original contributions presented in this study are included in the article/Supplementary Materials. Further inquiries can be directed to the corresponding author(s).

## Supplementary Materials

The following supporting information can be downloaded at: https://www.mdpi.com/article/10.3390/pharmaceutics18081029/s1.

## References

[1] Hong S.N. (2018). Genetic and epigenetic alterations of colorectal cancer. Intest. Res..

[2] Puneet, Kazmi H.R., Kumari S., Tiwari S., Khanna A., Narayan G. (2018). Epigenetic Mechanisms and Events in Gastric Cancer-Emerging Novel Biomarkers. Pathol. Oncol. Res..

[3] Diori Karidio I., Sanlier S.H. (2021). Reviewing cancer’s biology: An eclectic approach. J. Egypt. Natl. Cancer Inst..

[4] Derelanko M.J., Hollinger M.A. (2001). Handbook of Toxicology.

[5] Balkwill F.R., Capasso M., Hagemann T. (2012). The tumor microenvironment at a glance. J. Cell Sci..

[6] Wang R.-A., Li Z.-S., Zhang H.-Z., Zheng P.-J., Li Q.-L., Shi J.-G., Yan Q.-G., Ye J., Wang J.-B., Guo Y. (2013). Invasive cancers are not necessarily from preformed in situ tumours—An alternative way of carcinogenesis from misplaced stem cells. J. Cell. Mol. Med..

[7] Kontomanolis E.N., Koutras A., Syllaios A., Schizas D., Kalagasidou S., Pagkalos A., Alatzidou D., Kantari P., Ntounis T., Fasoulakis Z. (2021). Basic principles of molecular biology of cancer cell-Molecular cancer indicators. J. Buon.

[8] Li Q., Geng S., Luo H., Wang W., Mo Y.-Q., Luo Q., Wang L., Song G.-B., Sheng J.-P., Xu B. (2024). Signaling pathways involved in colorectal cancer: Pathogenesis and targeted therapy. Signal Transduct. Target. Ther..

[9] Jiao S., Peters U., Berndt S., Brenner H., Butterbach K., Caan B.J., Carlson C.S., Chan A.T., Chang-Claude J., Chanock S. (2014). Estimating the heritability of colorectal cancer. Hum. Mol. Genet..

[10] Sung H., Ferlay J., Siegel R.L., Laversanne M., Soerjomataram I., Jemal A., Bray F. (2021). Global Cancer Statistics 2020: GLOBOCAN Estimates of Incidence and Mortality Worldwide for 36 Cancers in 185 Countries. CA Cancer J. Clin..

[11] Fernandez-Rozadilla C., Timofeeva M., Chen Z., Law P., Thomas M., Schmit S., Díez-Obrero V., Hsu L., Fernandez-Tajes J., Palles C. (2023). Deciphering colorectal cancer genetics through multi-omic analysis of 100,204 cases and 154,587 controls of European and east Asian ancestries. Nat. Genet..

[12] Rumpold H., Niedersüß-Beke D., Heiler C., Falch D., Wundsam H.V., Metz-Gercek S., Piringer G., Thaler J. (2020). Prediction of mortality in metastatic colorectal cancer in a real-life population: A multicenter explorative analysis. BMC Cancer.

[13] Shin A.E., Giancotti F.G., Rustgi A.K. (2023). Metastatic colorectal cancer: Mechanisms and emerging therapeutics. Trends Pharmacol. Sci..

[14] Bray F., Laversanne M., Sung H., Ferlay J., Siegel R.L., Soerjomataram I., Jemal A. (2024). Global cancer statistics 2022: GLOBOCAN estimates of incidence and mortality worldwide for 36 cancers in 185 countries. CA Cancer J. Clin..

[15] Matsuda T., Fujimoto A., Igarashi Y. (2025). Colorectal Cancer: Epidemiology, Risk Factors, and Public Health Strategies. Digestion.

[16] Bray F., Ferlay J., Soerjomataram I., Siegel R.L., Torre L.A., Jemal A. (2018). Global cancer statistics 2018: GLOBOCAN estimates of incidence and mortality worldwide for 36 cancers in 185 countries. CA Cancer J. Clin..

[17] Dekker E., Tanis P.J., Vleugels J.L.A., Kasi P.M., Wallace M.B. (2019). Colorectal cancer. Lancet.

[18] Li J., Ma X., Chakravarti D., Shalapour S., DePinho R.A. (2021). Genetic and biological hallmarks of colorectal cancer. Genes Dev..

[19] Duan B., Zhao Y., Bai J., Wang J., Duan X., Luo X., Zhang R., Pu Y., Kou M., Lei J., Andres Morgado-Diaz J., Cellular and Molecular Oncobiology Program, Cellular Dynamic and Structure Group, National Cancer Institute-INCA (2022). Colorectal Cancer: An Overview. Gastrointestinal Cancers.

[20] Hofseth L.J., Hebert J.R., Chanda A., Chen H., Love B.L., Pena M.M., Murphy E.A., Sajish M., Sheth A., Buckhaults P.J. (2020). Early-onset colorectal cancer: Initial clues and current views. Nat. Rev. Gastroenterol. Hepatol..

[21] Roncucci L., Mariani F. (2015). Prevention of colorectal cancer: How many tools do we have in our basket?. Eur. J. Intern. Med..

[22] Maida M., Macaluso F.S., Ianiro G., Mangiola F., Sinagra E., Hold G., Maida C., Cammarota G., Gasbarrini A., Scarpulla G. (2017). Screening of colorectal cancer: Present and future. Expert Rev. Anticancer Ther..

[23] Murphy N., Moreno V., Hughes D.J., Vodicka L., Vodicka P., Aglago E.K., Gunter M.J., Jenab M. (2019). Lifestyle and dietary environmental factors in colorectal cancer susceptibility. Mol. Asp. Med..

[24] Hossain M.S., Karuniawati H., Jairoun A.A., Urbi Z., Ooi D.J., John A., Lim Y.C., Kibria K.M.K., Mohiuddin A.K.M., Ming L.C. (2022). Colorectal Cancer: A Review of Carcinogenesis, Global Epidemiology, Current Challenges, Risk Factors, Preventive and Treatment Strategies. Cancers.

[25] Mármol I., Sánchez-de-Diego C., Pradilla Dieste A., Cerrada E., Rodriguez Yoldi M. (2017). Colorectal Carcinoma: A General Overview and Future Perspectives in Colorectal Cancer. Int. J. Mol. Sci..

[26] Berner A.M., Murugaesu N. (2025). The Evolving Role of Genomics in Colorectal Cancer. Clin. Oncol..

[27] Al-Sohaily S., Biankin A., Leong R., Kohonen-Corish M., Warusavitarne J. (2012). Molecular pathways in colorectal cancer. J. Gastroenterol. Hepatol..

[28] Simon K. (2016). Colorectal cancer development and advances in screening. Clin. Interv. Aging.

[29] Poulogiannis G., Ichimura K., Hamoudi R.A., Luo F., Leung S.Y., Yuen S.T., Harrison D.J., Wyllie A.H., Arends M.J. (2010). Prognostic relevance of DNA copy number changes in colorectal cancer. J. Pathol..

[30] Ibrahim A.E.K., Arends M.J., Silva A.-L., Wyllie A.H., Greger L., Ito Y., Vowler S.L., Huang T.H.-M., Tavaré S., Murrell A. (2011). Sequential DNA methylation changes are associated with DNMT3B overexpression in colorectal neoplastic progression. Gut.

[31] Arends M.J. (2013). Pathways of colorectal carcinogenesis. Appl. Immunohistochem. Mol. Morphol..

[32] Müller M.F., Ibrahim A.E.K., Arends M.J. (2016). Molecular pathological classification of colorectal cancer. Virchows Arch..

[33] Nowak-Sliwinska P., Scapozza L., Ruiz i Altaba A. (2019). Drug repurposing in oncology: Compounds, pathways, phenotypes and computational approaches for colorectal cancer. Biochim. Biophys. Acta Rev. Cancer.

[34] Giampieri R., Cantini L., Giglio E., Bittoni A., Lanese A., Crocetti S., Pecci F., Copparoni C., Meletani T., Lenci E. (2020). Impact of Polypharmacy for Chronic Ailments in Colon Cancer Patients: A Review Focused on Drug Repurposing. Cancers.

[35] Jourdan J.-P., Bureau R., Rochais C., Dallemagne P. (2020). Drug repositioning: A brief overview. J. Pharm. Pharmacol..

[36] El Zarif T., Yibirin M., De Oliveira-Gomes D., Machaalani M., Nawfal R., Bittar G., Bahmad H.F., Bitar N. (2022). Overcoming Therapy Resistance in Colon Cancer by Drug Repurposing. Cancers.

[37] Kubota M., Shimizu M., Sakai H., Yasuda Y., Ohno T., Kochi T., Tsurumi H., Tanaka T., Moriwaki H. (2011). Renin-angiotensin system inhibitors suppress azoxymethane-induced colonic preneoplastic lesions in C57BL/KsJ-db/db obese mice. Biochem. Biophys. Res. Commun..

[38] Kedika R., Patel M., Pena Sahdala H.N., Mahgoub A., Cipher D., Siddiqui A.A. (2011). Long-term use of angiotensin converting enzyme inhibitors is associated with decreased incidence of advanced adenomatous colon polyps. J. Clin. Gastroenterol..

[39] Chan A.T., Ogino S., Fuchs C.S. (2007). Aspirin and the risk of colorectal cancer in relation to the expression of COX-2. N. Engl. J. Med..

[40] Saber M.M., Galal M.A., Ain-Shoka A.A., Shouman S.A. (2016). Combination of metformin and 5-aminosalicylic acid cooperates to decrease proliferation and induce apoptosis in colorectal cancer cell lines. BMC Cancer.

[41] Bertagnolli M.M., Eagle C.J., Zauber A.G., Redston M., Solomon S.D., Kim K., Tang J., Rosenstein R.B., Wittes J., Corle D. (2006). Celecoxib for the prevention of sporadic colorectal adenomas. N. Engl. J. Med..

[42] Xu X.-T., Hu W.-T., Zhou J.-Y., Tu Y. (2017). Celecoxib enhances the radiosensitivity of HCT116 cells in a COX-2 independent manner by up-regulating BCCIP. Am. J. Transl. Res..

[43] Alzahrani S.M., Al Doghaither H.A., Alkhatabi H.A., Basabrain M.A., Pushparaj P.N. (2025). Investigating the Potential of Propranolol as an Anti-Tumor Agent in Colorectal Cancer Cell Lines. Int. J. Mol. Sci..

[44] El Menshawe S.F., Shalaby K., Elkomy M.H., Aboud H.M., Ahmed Y.M., Abdelmeged A.A., Elkarmalawy M., Abou Alazayem M.A., El Sisi A.M. (2024). Repurposing celecoxib for colorectal cancer targeting via pH-triggered ultra-elastic nanovesicles: Pronounced efficacy through up-regulation of Wnt/β-catenin pathway in DMH-induced tumorigenesis. Int. J. Pharm. X.

[45] Liñares-Blanco J., Munteanu C.R., Pazos A., Fernandez-Lozano C. (2020). Molecular docking and machine learning analysis of Abemaciclib in colon cancer. BMC Mol. Cell Biol..

[46] Matthews H., Hanison J., Nirmalan N. (2016). “Omics”-Informed Drug and Biomarker Discovery: Opportunities, Challenges and Future Perspectives. Proteomes.

[47] Sun J., Zhao J., Jiang F., Wang L., Xiao Q., Han F., Chen J., Yuan S., Wei J., Larsson S.C. (2023). Identification of novel protein biomarkers and drug targets for colorectal cancer by integrating human plasma proteome with genome. Genome Med..

[48] Cornish A.J., Gruber A.J., Kinnersley B., Chubb D., Frangou A., Caravagna G., Noyvert B., Lakatos E., Wood H.M., Thorn S. (2024). The genomic landscape of 2023 colorectal cancers. Nature.

[49] Laskar R.S., Qu C., Huyghe J.R., Harrison T., Hayes R.B., Cao Y., Campbell P.T., Steinfelder R., Talukdar F.R., Brenner H. (2024). Genome-wide association studies and Mendelian randomization analyses provide insights into the causes of early-onset colorectal cancer. Ann. Oncol..

[50] Zhu Q., Hulen D., Liu T., Clarke M. (1997). The cluA- mutant of Dictyostelium identifies a novel class of proteins required for dispersion of mitochondria. Proc. Natl. Acad. Sci. USA.

[51] Gao J., Schatton D., Martinelli P., Hansen H., Pla-Martin D., Barth E., Becker C., Altmueller J., Frommolt P., Sardiello M. (2014). CLUH regulates mitochondrial biogenesis by binding mRNAs of nuclear-encoded mitochondrial proteins. J. Cell Biol..

[52] Wakim J., Goudenege D., Perrot R., Gueguen N., Desquiret-Dumas V., Chao de la Barca J.M., Dalla Rosa I., Manero F., Le Mao M., Chupin S. (2017). CLUH couples mitochondrial distribution to the energetic and metabolic status. J. Cell Sci..

[53] Schatton D., Pla-Martin D., Marx M.-C., Hansen H., Mourier A., Nemazanyy I., Pessia A., Zentis P., Corona T., Kondylis V. (2017). CLUH regulates mitochondrial metabolism by controlling translation and decay of target mRNAs. J. Cell Biol..

[54] Zhang D., Wang W., Xiang B., Li N., Huang S., Zhou W., Sun Y., Wang X., Ma J., Li G. (2013). Reduced succinate dehydrogenase B expression is associated with growth and de-differentiation of colorectal cancer cells. Tumor Biol..

[55] Luo Y., Ma J., Lu W. (2020). The Significance of Mitochondrial Dysfunction in Cancer. Int. J. Mol. Sci..

[56] Singh S., Gaur A., Sharma R.K., Kumari R., Prakash S., Kumari S., Chaudhary A.D., Prasun P., Pant P., Hunkler H. (2023). Musashi-2 causes cardiac hypertrophy and heart failure by inducing mitochondrial dysfunction through destabilizing Cluh and Smyd1 mRNA. Basic Res. Cardiol..

[57] Kharin L., Bychkov I., Karnaukhov N., Voloshin M., Fazliyeva R., Deneka A., Frantsiyants E., Kit O., Golemis E., Boumber Y. (2021). Prognostic role and biologic features of Musashi-2 expression in colon polyps and during colorectal cancer progression. PLoS ONE.

[58] Pettem K.L., Yokomaku D., Luo L., Linhoff M.W., Prasad T., Connor S.A., Siddiqui T.J., Kawabe H., Chen F., Zhang L. (2013). The specific α-neurexin interactor calsyntenin-3 promotes excitatory and inhibitory synapse development. Neuron.

[59] Lu Z., Wang Y., Chen F., Tong H., Reddy M.V.V.V.S., Luo L., Seshadrinathan S., Zhang L., Holthauzen L.M.F., Craig A.M. (2014). Calsyntenin-3 molecular architecture and interaction with neurexin 1α. J. Biol. Chem..

[60] Um J.W., Pramanik G., Ko J.S., Song M.-Y., Lee D., Kim H., Park K.-S., Südhof T.C., Tabuchi K., Ko J. (2014). Calsyntenins function as synaptogenic adhesion molecules in concert with neurexins. Cell Rep..

[61] Guo J., Huang S., Yi Q., Liu N., Cui T., Duan S., Chen J., Li J., Li J., Wang L. (2023). Hepatic Clstn3 Ameliorates Lipid Metabolism Disorders in High Fat Diet-Induced NAFLD through Activation of FXR. ACS Omega.

[62] Chu Y., Lai Y.-H., Lee M.-C., Yeh Y.-J., Wu Y.-K., Tsao W., Huang C.-Y., Wu S. (2017). Calsyntenin-1, clusterin and neutrophil gelatinase-associated lipocalin are candidate serological biomarkers for lung adenocarcinoma. Oncotarget.

[63] Kim S.-J., Jeong Y.T., Jeong S.R., Park M., Go H.S., Kim M.Y., Seong J.K., Kim K.W., Seo J.T., Kim C.H. (2020). Neural regulation of energy and bone homeostasis by the synaptic adhesion molecule Calsyntenin-3. Exp. Mol. Med..

[64] Bai N., Lu X., Jin L., Alimujiang M., Ma J., Hu F., Xu Y., Sun J., Xu J., Zhang R. (2022). *CLSTN3* gene variant associates with obesity risk and contributes to dysfunction in white adipose tissue. Mol. Metab..

[65] Meng J.Y., Kataoka H., Itoh H., Koono M. (2001). Amyloid beta protein precursor is involved in the growth of human colon carcinoma cell in vitro and in vivo. Int. J. Cancer.

[66] Gunshin H., Mackenzie B., Berger U.V., Gunshin Y., Romero M.F., Boron W.F., Nussberger S., Gollan J.L., Hediger M.A. (1997). Cloning and characterization of a mammalian proton-coupled metal-ion transporter. Nature.

[67] Tian L., Li X., Lai H., Sun T., Li X., Wu L., Wu C., Yao S., Ren Y., He S. (2023). SLC11A2: A promising biomarker and therapeutic target in ovarian cancer. Sci. Rep..

[68] Kaushik V., Yakisich J.S., Kumar A., Azad N., Iyer A.K.V. (2018). Ionophores: Potential Use as Anticancer Drugs and Chemosensitizers. Cancers.

[69] Merk K., Mattsson B., Mattsson A., Holm G., Gullbring B., Björkholm M. (1990). The incidence of cancer among blood donors. Int. J. Epidemiol..

[70] Nelson R.L. (2001). Iron and colorectal cancer risk: Human studies. Nutr. Rev..

[71] Zacharski L.R., Chow B.K., Howes P.S., Shamayeva G., Baron J.A., Dalman R.L., Malenka D.J., Ozaki C.K., Lavori P.W. (2008). Decreased cancer risk after iron reduction in patients with peripheral arterial disease: Results from a randomized trial. J. Natl. Cancer Inst..

[72] Osborne N.J., Gurrin L.C., Allen K.J., Constantine C.C., Delatycki M.B., McLaren C.E., Gertig D.M., Anderson G.J., Southey M.C., Olynyk J.K. (2010). *HFE C282Y* homozygotes are at increased risk of breast and colorectal cancer. Hepatology.

[73] Cross A.J., Ferrucci L.M., Risch A., Graubard B.I., Ward M.H., Park Y., Hollenbeck A.R., Schatzkin A., Sinha R. (2010). A large prospective study of meat consumption and colorectal cancer risk: An investigation of potential mechanisms underlying this association. Cancer Res..

[74] Brookes M.J., Hughes S., Turner F.E., Reynolds G., Sharma N., Ismail T., Berx G., McKie A.T., Hotchin N., Anderson G.J. (2006). Modulation of iron transport proteins in human colorectal carcinogenesis. Gut.

[75] Xue X., Taylor M., Anderson E., Hao C., Qu A., Greenson J.K., Zimmermann E.M., Gonzalez F.J., Shah Y.M. (2012). Hypoxia-inducible factor-2α activation promotes colorectal cancer progression by dysregulating iron homeostasis. Cancer Res..

[76] Xue X., Shah Y.M. (2013). Intestinal iron homeostasis and colon tumorigenesis. Nutrients.

[77] Seril D.N., Liao J., Yang G.-Y., Yang C.S. (2003). Oxidative stress and ulcerative colitis-associated carcinogenesis: Studies in humans and animal models. Carcinogenesis.

[78] Heath A.P., Ferretti V., Agrawal S., An M., Angelakos J.C., Arya R., Bajari R., Baqar B., Barnowski J.H.B., Burt J. (2021). The NCI Genomic Data Commons. Nat. Genet..

[79] Mi H., Huang X., Muruganujan A., Tang H., Mills C., Kang D., Thomas P.D. (2017). PANTHER version 11: Expanded annotation data from Gene Ontology and Reactome pathways, and data analysis tool enhancements. Nucleic Acids Res..

[80] Manfredi M., Savojardo C., Martelli P.L., Casadio R. (2022). E-SNPs&GO: Embedding of protein sequence and function improves the annotation of human pathogenic variants. Bioinformatics.

[81] Masica D.L., Douville C., Tokheim C., Bhattacharya R., Kim R., Moad K., Ryan M.C., Karchin R. (2017). CRAVAT 4: Cancer-Related Analysis of Variants Toolkit. Cancer Res..

[82] Tate J.G., Bamford S., Jubb H.C., Sondka Z., Beare D.M., Bindal N., Boutselakis H., Cole C.G., Creatore C., Dawson E. (2019). COSMIC: The Catalogue Of Somatic Mutations In Cancer. Nucleic Acids Res..

[83] Waterhouse A., Bertoni M., Bienert S., Studer G., Tauriello G., Gumienny R., Heer F.T., de Beer T.A.P., Rempfer C., Bordoli L. (2018). SWISS-MODEL: Homology modelling of protein structures and complexes. Nucleic Acids Res..

[84] DeLano W.L. (2002). PyMOL: An Open-Source Molecular Graphics Tool. CCP4 Newsl. Protein Crystallogr..

[85] Wang Z., Lipshutz A., Martínez de la Torre C., Trzeciak A.J., Liu Z.-L., Miranda I.C., Lazarov T., Codo A.C., Romero-Pichardo J.E., Nair A. (2025). Early life high fructose impairs microglial phagocytosis and neurodevelopment. Nature.

[86] Irwin J.J., Tang K.G., Young J., Dandarchuluun C., Wong B.R., Khurelbaatar M., Moroz Y.S., Mayfield J., Sayle R.A. (2020). ZINC20—A Free Ultralarge-Scale Chemical Database for Ligand Discovery. J. Chem. Inf. Model..

[87] Dallakyan S., Olson A.J. (2015). Small-molecule library screening by docking with PyRx. Chem. Biol..

[88] NIST (2012). NIST Engineering Statistics Handbook.

[89] Ye J., Coulouris G., Zaretskaya I., Cutcutache I., Rozen S., Madden T.L. (2012). Primer-BLAST: A tool to design target-specific primers for polymerase chain reaction. BMC Bioinform..

[90] Siegel R.L., Kratzer T.B., Giaquinto A.N., Sung H., Jemal A. (2025). Cancer statistics, 2025. CA Cancer J. Clin..

[91] Zhou J., Lin H., Zheng J., Wang X. (2025). Integrative eQTL and Mendelian randomization analysis reveals key genes in colorectal cancer. Discov. Oncol..

[92] Kozako T., Soeda S., Yoshimitsu M., Arima N., Kuroki A., Hirata S., Tanaka H., Imakyure O., Tone N., Honda S.-I. (2016). Angiotensin II type 1 receptor blocker telmisartan induces apoptosis and autophagy in adult T-cell leukemia cells. FEBS Open Bio.

[93] Grahovac J., Srdić-Rajić T., Francisco Santibañez J., Pavlović M., Čavić M., Radulović S. (2019). Telmisartan induces melanoma cell apoptosis and synergizes with vemurafenib in vitro by altering cell bioenergetics. Cancer Biol. Med..

[94] Fujita N., Fujita K., Iwama H., Kobara H., Fujihara S., Chiyo T., Namima D., Yamana H., Kono T., Takuma K. (2020). Antihypertensive drug telmisartan suppresses the proliferation of gastric cancer cells in vitro and in vivo. Oncol. Rep..

[95] Muradi Muhar A., Velaro A.J., Prananda A.T., Nugraha S.E., Halim P., Syahputra R.A. (2025). Precision medicine in colorectal cancer: Genomics profiling and targeted treatment. Front. Pharmacol..

[96] Cortés-Ciriano I., Gulhan D.C., Lee J.J.-K., Melloni G.E.M., Park P.J. (2022). Computational analysis of cancer genome sequencing data. Nat. Rev. Genet..

[97] Jauhri M., Bhatnagar A., Gupta S., Bp M., Minhas S., Shokeen Y., Aggarwal S. (2017). Prevalence and coexistence of KRAS, BRAF, PIK3CA, NRAS, TP53, and APC mutations in Indian colorectal cancer patients: Next-generation sequencing-based cohort study. Tumor Biol..

[98] Sclafani F., Wilson S.H., Cunningham D., Gonzalez De Castro D., Kalaitzaki E., Begum R., Wotherspoon A., Capdevila J., Glimelius B., Roselló S. (2020). Analysis of KRAS, NRAS, BRAF, PIK3CA and TP53 mutations in a large prospective series of locally advanced rectal cancer patients. Int. J. Cancer.

[99] Marbun V.M.G., Erlina L., Lalisang T.J.M. (2022). Genomic landscape of pathogenic mutation of APC, KRAS, TP53, PIK3CA, and MLH1 in Indonesian colorectal cancer. PLoS ONE.

[100] Afrăsânie V.-A., Marinca M.-V., Gafton B., Alexa-Stratulat T., Rusu A., Froicu E.-M., Sur D., Lungulescu C.V., Popovici L., Lefter A.-V. (2023). Clinical, Pathological and Molecular Insights on KRAS, NRAS, BRAF, PIK3CA and TP53 Mutations in Metastatic Colorectal Cancer Patients from Northeastern Romania. Int. J. Mol. Sci..

[101] Feng Y., Spezia M., Huang S., Yuan C., Zeng Z., Zhang L., Ji X., Liu W., Huang B., Luo W. (2018). Breast cancer development and progression: Risk factors, cancer stem cells, signaling pathways, genomics, and molecular pathogenesis. Genes Dis..

[102] Malone E.R., Oliva M., Sabatini P.J.B., Stockley T.L., Siu L.L. (2020). Molecular profiling for precision cancer therapies. Genome Med..

[103] Naito Y., Aburatani H., Amano T., Baba E., Furukawa T., Hayashida T., Hiyama E., Ikeda S., Kanai M., Kato M. (2021). Clinical practice guidance for next-generation sequencing in cancer diagnosis and treatment (edition 2.1). Int. J. Clin. Oncol..

[104] Dharani S., Kamaraj R. (2024). A Review of the Regulatory Challenges of Personalized Medicine. Cureus.

[105] Dienstmann R., Salazar R., Tabernero J. (2014). The Evolution of Our Molecular Understanding of Colorectal Cancer: What We Are Doing Now, What the Future Holds, and How Tumor Profiling Is Just the Beginning. Am. Soc. Clin. Oncol. Educ. Book.

[106] Underwood P.W., Ruff S.M., Pawlik T.M. (2024). Update on Targeted Therapy and Immunotherapy for Metastatic Colorectal Cancer. Cells.

[107] Tang Y.-L., Li D.-D., Duan J.-Y., Sheng L.-M., Wang X. (2023). Resistance to targeted therapy in metastatic colorectal cancer: Current status and new developments. World J. Gastroenterol..

[108] Manco M., Ala U., Cantarella D., Tolosano E., Medico E., Altruda F., Fagoonee S., Manco M., Ala U., Cantarella D. (2021). The RNA-Binding Protein ESRP1 Modulates the Expression of RAC1b in Colorectal Cancer Cells. Cancers.

[109] Li C., Yin Y., Tao R., Lin Y., Wang T., Shen Q., Li R., Tao K., Liu W. (2023). ESRP1-driven alternative splicing of CLSTN1 inhibits the metastasis of gastric cancer. Cell Death Discov..

[110] Christou N., Perraud A., Blondy S., Jauberteau M.-O., Battu S., Mathonnet M. (2017). E-cadherin: A potential biomarker of colorectal cancer prognosis (Review). Oncol. Lett..

[111] Uhlen M., Zhang C., Lee S., Sjöstedt E., Fagerberg L., Bidkhori G., Benfeitas R., Arif M., Liu Z., Edfors F. (2017). A pathology atlas of the human cancer transcriptome. Science.

[112] Jin H., Zhang C., Zwahlen M., von Feilitzen K., Karlsson M., Shi M., Yuan M., Song X., Li X., Yang H. (2023). Systematic transcriptional analysis of human cell lines for gene expression landscape and tumor representation. Nat. Commun..

[113] Hu X., Harvey S.E., Zheng R., Lyu J., Grzeskowiak C.L., Powell E., Piwnica-Worms H., Scott K.L., Cheng C. (2020). The RNA-binding protein AKAP8 suppresses tumor metastasis by antagonizing EMT-associated alternative splicing. Nat. Commun..

[114] Xue X., Ramakrishnan S.K., Weisz K., Triner D., Xie L., Attili D., Pant A., Győrffy B., Zhan M., Carter-Su C. (2016). Iron Uptake via DMT1 Integrates Cell Cycle with JAK-STAT3 Signaling to Promote Colorectal Tumorigenesis. Cell Metab..

[115] Tajima S., Ikeda Y., Enomoto H., Imao M., Horinouchi Y., Izawa-Ishizawa Y., Kihira Y., Miyamoto L., Ishizawa K., Tsuchiya K. (2015). Angiotensin II alters the expression of duodenal iron transporters, hepatic hepcidin, and body iron distribution in mice. Eur. J. Nutr..

[116] Ahmed D., Eide P.W., Eilertsen I.A., Danielsen S.A., Eknæs M., Hektoen M., Lind G.E., Lothe R.A. (2013). Epigenetic and genetic features of 24 colon cancer cell lines. Oncogenesis.

[117] Parvathaneni V., Kulkarni N.S., Muth A., Gupta V. (2019). Drug repurposing: A promising tool to accelerate the drug discovery process. Drug Discov. Today.

[118] Pushpakom S., Iorio F., Eyers P.A., Escott K.J., Hopper S., Wells A., Doig A., Guilliams T., Latimer J., McNamee C. (2019). Drug repurposing: Progress, challenges and recommendations. Nat. Rev. Drug Discov..

[119] Fong W., To K.K.W. (2019). Drug repurposing to overcome resistance to various therapies for colorectal cancer. Cell. Mol. Life Sci. CMLS.

[120] Roberts A.W., Davids M.S., Pagel J.M., Kahl B.S., Puvvada S.D., Gerecitano J.F., Kipps T.J., Anderson M.A., Brown J.R., Gressick L. (2016). Targeting BCL2 with Venetoclax in Relapsed Chronic Lymphocytic Leukemia. N. Engl. J. Med..

[121] Lok S.W., Whittle J.R., Vaillant F., Teh C.E., Lo L.L., Policheni A.N., Bergin A.R.T., Desai J., Ftouni S., Gandolfo L.C. (2019). A Phase Ib Dose-Escalation and Expansion Study of the BCL2 Inhibitor Venetoclax Combined with Tamoxifen in ER and BCL2-Positive Metastatic Breast Cancer. Cancer Discov..

[122] Lochmann T.L., Floros K.V., Naseri M., Powell K.M., Cook W., March R.J., Stein G.T., Greninger P., Maves Y.K., Saunders L.R. (2018). Venetoclax Is Effective in Small-Cell Lung Cancers with High BCL-2 Expression. Clin. Cancer Res..

[123] Zhao D., Ding X., Peng J., Zheng Y., Zhang S. (2005). Prognostic significance of bcl-2 and p53 expression in colorectal carcinoma. J. Zhejiang Univ. Sci. B.

[124] Wu D.-W., Huang C.-C., Chang S.-W., Chen T.-H., Lee H. (2015). Bcl-2 stabilization by paxillin confers 5-fluorouracil resistance in colorectal cancer. Cell Death Differ..

[125] Qiu Z., Qiu S., Mao W., Lin W., Peng Q., Chang H. (2023). LOXL2 reduces 5-FU sensitivity through the Hedgehog/BCL2 signaling pathway in colorectal cancer. Exp. Biol. Med..

[126] Wei R., Zhong S., Qiao L., Guo M., Shao M., Wang S., Jiang B., Yang Y., Gu C. (2020). Steroid 5α-Reductase Type I Induces Cell Viability and Migration via Nuclear Factor-κB/Vascular Endothelial Growth Factor Signaling Pathway in Colorectal Cancer. Front. Oncol..

[127] Andriole G.L., Bostwick D.G., Brawley O.W., Gomella L.G., Marberger M., Montorsi F., Pettaway C.A., Tammela T.L., Teloken C., Tindall D.J. (2010). Effect of dutasteride on the risk of prostate cancer. N. Engl. J. Med..

[128] Refaat B., Aslam A., Idris S., Almalki A.H., Alkhaldi M.Y., Asiri H.A., Almaimani R.A., Mujalli A., Minshawi F., Alamri S.A. (2023). Profiling estrogen, progesterone, and androgen receptors in colorectal cancer in relation to gender, menopausal status, clinical stage, and tumour sidedness. Front. Endocrinol..

[129] Michel M.C., Foster C., Brunner H.R., Liu L. (2013). A systematic comparison of the properties of clinically used angiotensin II type 1 receptor antagonists. Pharmacol. Rev..

[130] Li J., Chen L., Yu P., Liu B., Zhu J., Yang Y., Li J., Chen L., Yu P., Liu B. (2014). Telmisartan Exerts Anti-Tumor Effects by Activating Peroxisome Proliferator-Activated Receptor-γ in Human Lung Adenocarcinoma A549 Cells. Molecules.

[131] Atiyah R., Kamil R., Abdullah T., Barqee A. (2025). Novel Anti-proliferative Application of Telmisartan and Candesartan: Synergistic Repurposing Strategy Against Colorectal Cancer. Al Anbar Med. J..

[132] El-Baz A.M., El-Mahmoudy A.A., Saber S., ElRakaiby M.T. (2025). The coadministration of Lactobacillus probiotic augments the antitumor effect of telmisartan in rats. AMB Express.

