# Supplementary material for: Telmisartan Repurposing Targets Novel Biomarkers for Precision Colorectal Cancer Therapy
Source: Pharmaceutics. 2026 Aug 20;18(8):1029. doi: 10.3390/pharmaceutics18081029 (PMC13516443; doi:10.3390/pharmaceutics18081029)
Supplement: Supplementary file 1 [file pharmaceutics-18-01029-s001.zip › pharmaceutics-4410864-supplementary.pdf]

Article

Telmisartan Repurposing Targets Novel Biomarkers for Precision Colorectal Cancer Therapy

Sarah Hunachagi [1], Hoor Hashim Alqudihi [1], Sayed AbdulAzeez [2,*], J. Francis Borgio [2,3,] and Dana Almohazey [4,*]

Supplementary Materials:

Supplementary Figures:

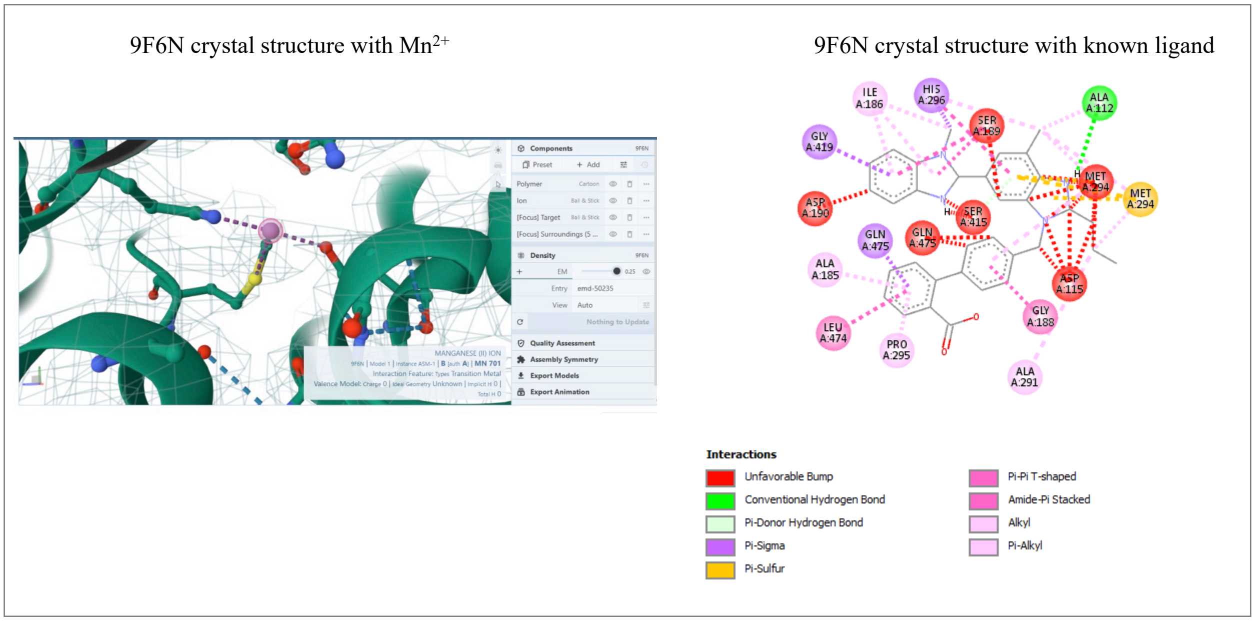

Figure S1: Structural analysis of the 9F6N crystal structure with Mn2+ and a known ligand. Left: The 9F6N crystal structure containing a co-crystallized Mn2+ ion (represented as a purple sphere) located in the active site. Right: 2D interaction diagram of the 9F6N crystal structure with a known ligand, mapping residue interactions.

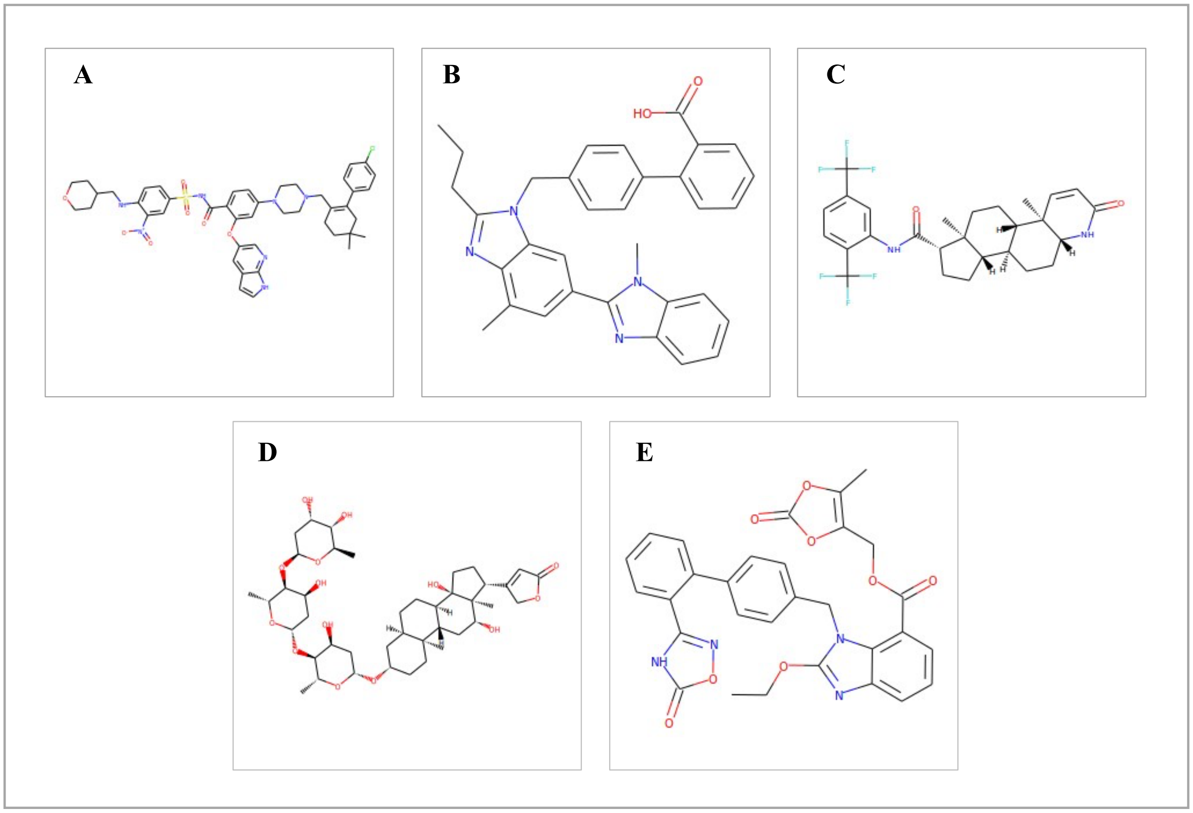

Figure S2. Chemical structures of selected drugs proposed for repurposing; **A:** Venetoclax (ZINC000150338755); **B:** Telmisartan (ZINC000001530886); **C:** Dutasteride/ Avodart (ZINC000003932831); **D:** Digoxin (ZINC242548690); **E:** Azilsartan medoxomil (ZINC14210642).

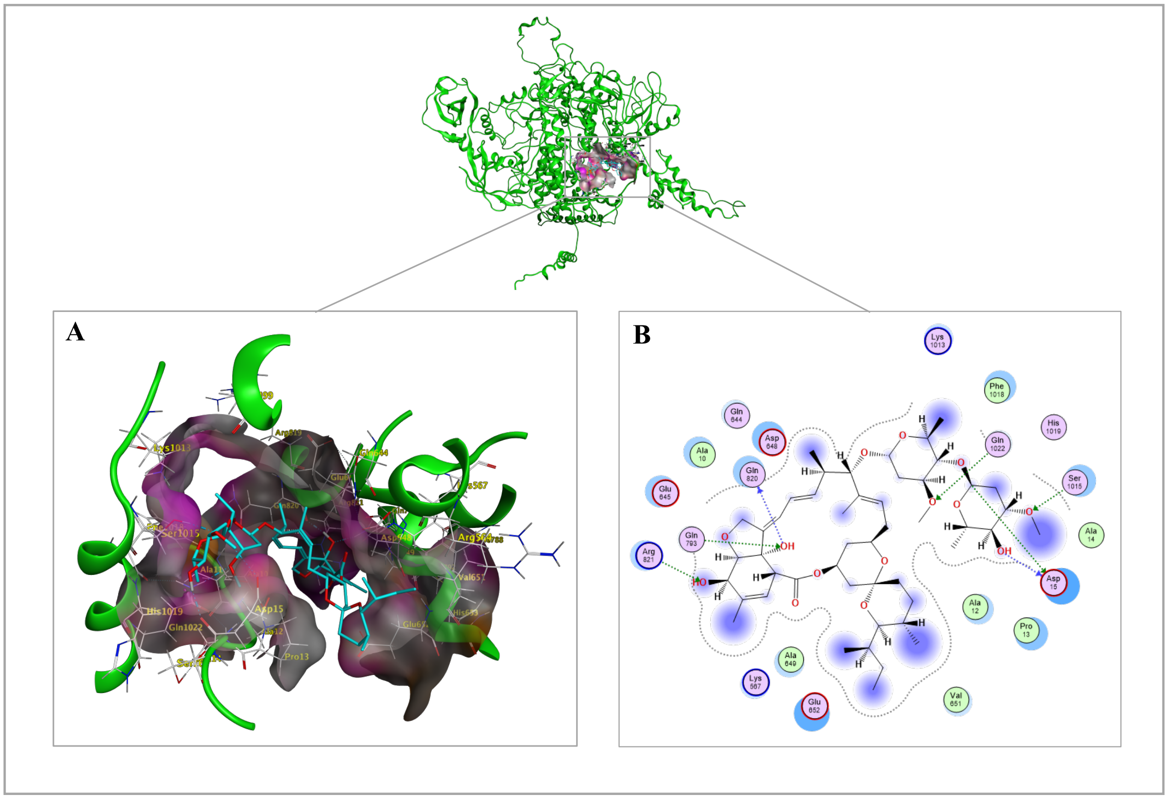

Figure S3. Protein-drug complex of CLUH and Venetoclax; A and B illustrate three and two dimensional and complexes, respectively. Hydrogen bonds are represented by green dashed lines.

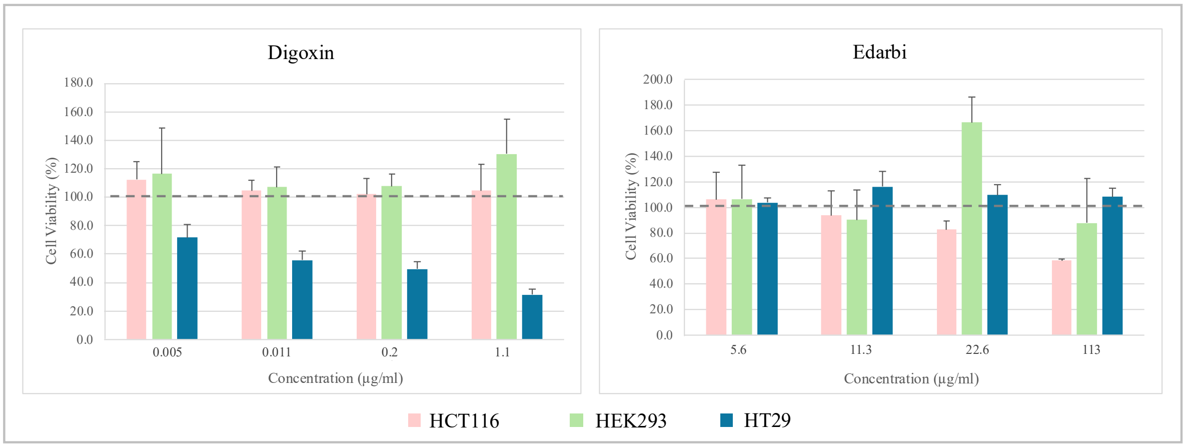

Figure S4. *In vitro* Cytotoxicity Profile of Digoxin and Azilsartan medoxomil. The colored bars represent mean ± SD of the corresponding dose; Dashed line at 100% represents the non-treatment control (NTC) threshold.

Supplementary Tables:

Table S1: Multi-database validation and expression analysis of CLUH, CLSTN3 and SLC11A2 in colorectal cancer (CRC).

Validation Parameter	CLUH	CLSTN3	SLC11A2
GEPIA2 (TCGA + GTEx) Expression	Downregulated in both COAD and READ compared with normal tissues	No marked differential expression; slight reduction observed in COAD	Upregulated in COAD; no marked difference in READ
CPTAC (UALCAN) Protein Expression	No significant difference between normal and primary tumors (P = 0.102)	Protein not identified in the CPTAC colon cancer dataset	Significantly increased protein expression in primary tumors compared with normal tissues (P = 1.57×10^{-9})
cBioPortal Cohort	23 CRC studies; 6,755 patients (7,001 samples)	23 CRC studies; 6,755 patients (7,001 samples)	23 CRC studies; 6,755 patients (7,001 samples)
Genomic Alteration Frequency	220/6,755 patients (3%)	175/6,755 patients (3%)	88/6,755 patients (1%)
Predominant Genomic Alteration	Deep deletions	Amplifications, followed by deep deletions	Amplifications, followed by deep deletions
Overall Survival (GEPIA2)	Not significant (Log-rank P = 0.41; HR = 0.83)	Not significant (Log-rank P = 0.11; HR = 1.40)	Not significant (Log-rank P = 0.49; HR = 0.86)
Disease-Free Survival (GEPIA2)	Not significant (Log-rank P = 0.78; HR = 0.94)	Significantly associated with poorer DFS (Log-rank P = 0.033; HR = 1.60)	Not significant (Log-rank P = 0.26; HR = 0.78)

Table S2. PolyPhen-2 scores and qualitative predictions.

PolyPhen-2 Value	Qualitative Prediction
>0.908	"Probably Damaging"
>0.446 and ≤ 0.908	"Possibly Damaging"
≤ 0.446	"Benign"
unknown	"Unknown"

Table S3. PANTHER classification based on Position-Specific Evolutionary Preservation (PSEP) scores.

PANTHER Classification	PSEP Score (Millions of Years)	Pdel Score (Probability)
Probably Damaging	> 450	> 0.75
Possibly Damaging	200 - 450	0.25 – 0.75
Probably Benign	< 200	< 0.25

Table S4. Working Concentrations of the Four Doses for Each Drug.

Drug Name	Dose 1 (μg/ml)	Dose 2 (μg/ml)	Dose 3 (μg/ml)	Dose 4 (μg/ml)
Venetoclax	2.1	4.3	8.7	43
Telmisartan	4.1	8.3	33	165
Dutasteride	0.02	0.08	0.32	0.8
Digoxin	0.005	0.01	0.2	1.1
Azilsartan medoxomil	5.6	11.3	22.6	113
Cisplatin	1.25	2.5	5	10

Table S5. EC_{50} and EC_{20} Concentrations for HCT116, HT29 and HEK293.

Cell Line	EC_{50} (μg/mL)	EC_{20} (μg/mL)
HCT116	14.77	2.95
HT29	18.98	3.79
HEK293	6.185	1.23

Table S6. Components of Master Mix for cDNA synthesis.

Reagents	Volume (μL)
5X Vilo Reaction Mix	4
10X Superscript Enzyme Mix	2
DEPC-Treated H_2O	4
**Total**	**10**

Table S7. Cycling Conditions for cDNA Synthesis.

Temperature	Time
25 °C	10 min
42 °C	60 min
85 °C	5 min

Table S8. Expression primers with their amplicon sizes.

	Name of the Primer	Sequence (5′ to 3′)	Amplicon (bp)
1	CLUHexF	AATCGGCATTGGAGAACTCATCACC	298
2	CLUHexR	TGGATCCCTGTTTTCAGCGAGATCT	
3	CLSTN3exF	AGACCTGTGATGAACCACTCTGGAA	344
4	CLSTN3exR	AGTTACGCCATGCTTCATCCAGAAG	
5	SLC11A2exF	AATCCGATTTGCAGTCTGGAGCAG	306
6	SLC11A2exR	TATCTGCAATGGTGATGAGAACGCC	

7	ACTBexF	AGAGCTACGAGCTGCCTGAC	184
8	ACTBexR	AGCACTGTGTTGGCGTACAG	
9	GAPDHexF	ATTCCACCCATGGCAAATTCCATG	306
10	GAPDHexR	AGGAGGCATTGCTGATGATCTTGAG	

Table S9. RT-PCR Master mix preparation using half-formula.

**Components**	**Volume (μL)**
Power SYBR™ Green PCR Master Mix (2X)	5
Forward primer	1
Reverse primer	1
PCR- grade water	1
**Total**	**8**

Table S10. Thermal cycling conditions for RT-PCR.

**Step**	**Temperature**	**Duration**	
UDG activation	50 °C	2 min	
DNA polymerase activation	95 °C	10 sec	
Denaturation	95 °C	15 sec	x40 cycles
Annealing and Extension	60 °C	1 min	
Melt curve	95 °C	15 sec	
	60 °C	1 min	
	95 °C	15 sec	

Table S11. List of Mutations reported in Large Intestine from Gene of Interest.

**Gene Name**	**Amino Acid Change**	**COSMIC ID**	**COSMIC Variant Count (Tissue)**
CLUH	T483M	COSM1381927	Large intestine (1); CNS (1); skin (1)
	T827M	COSM1381912	Large intestine (1)
	A444V	COSM4064837	Large intestine (1); stomach (1)
	R117C	COSM1381938	Large intestine (1); stomach (1)
	R276H	COSM1211987	Large intestine (1)
	L1029P	COSM1381900	Large intestine (1)
	R1206H	COSM1381893	Large intestine (1)
	R560C	COSM5158945	Large intestine (1)
	A391V	COSM1381929	Large intestine (1)
	P1298L	COSM1381889	Large intestine (1)
	D909N	COSM1211989	Large intestine (1)

	A1081T	COSM1381895	Large intestine (1)
	R787H	COSM1381914	Large intestine (2)
	A1261V	COSM2799511	Large intestine (1)
	R133C	COSM2799725	Large intestine (1); stomach (1)
	Y459H	COSM2799659	Large intestine (1)
	R532Q	COSM1381925	Large intestine (1)
	D442TWRPTWRPPTT*	COSM2799673	Large intestine (2); prostate (1)
	P572RTSTSCPCLARSCLRN APAPASPAPTGTSSAACAR SWWTPSWSTGTSSL*	COSM4615260	Large intestine (1); stomach (1)
	P878RGLQITQPGLS*	COSM1381910	Large intestine (1)
	P973PRPRMPSISS RAGRPKCSRAS*	COSM1381907	Large intestine (1)
	Q67RRWCRRFTRCSWTGR TRVTAPASHCTWMATCW TTSRSCAASRGCRRALCC VWWKSRTRCVRPASTCA MSETCSRAWTHPMPSTG LTATPCPS*	COSM4695360	Large intestine (1)
CLSTN3	D149N	COSM3417119	Large intestine (1)
	A652T	COSM1639245	Large intestine (1); stomach (1)
	A860T	COSM1364146	Large intestine (1); stomach (1)
	R887H	COSM1364148	Large intestine (1)
	E266K	COSM1364142	Large intestine (1)
	L473H	COSM1364143	Large intestine (1)
	A338A	COSM297101	Large intestine (1)
	E962K	COSM943369	Large intestine (1); endometrium (1)
	V595M	COSM300143	Large intestine (1)
	A91V	COSM268904	Large intestine (1)
	T589T	COSM1364144	Large intestine (1)
	R959H	COSM1201464	Large intestine (3)
	C867C	COSM1364147	Large intestine (1)
	R443H	COSM549819	Large intestine (1); stomach (1)
	R81W	COSM4672948	Large intestine (1)
	T963TPHTAT	COSM1364149	Large intestine (2)
	A652APRSC*	COSM4612326	Large intestine (1)
SLC11A2	R278H	COSM1362304	Large intestine (2)
	A77T	COSM32671	Large intestine (2)
	R437C	COSM1362303	Large intestine (1)

F240LAFSSLLW PSHLDMSMLQ*	COSM1362305	Large intestine (6)
